# Advancements in nanomaterials for the treatment and management of vascular surgeries: from drug delivery to biomedical implants

**DOI:** 10.3389/fbioe.2026.1788897

**Published:** 2026-03-24

**Authors:** Jianxin Dong, Ming Sun, Yanmei Li, Zhilei Xie

**Affiliations:** 1 Department of Vascular Surgery, Yantai Mountain Hospital, Yantai, Shandong, China; 2 Vascular Surgery, The Affiliated Taian City Central Hospital of Qingdao University, Taian, Shandong, China

**Keywords:** biomedical implants, drug delivery, nanomaterial, nanomedicine, nanotechnology, vascular surgery

## Abstract

Vascular surgery is an important procedure that is carried out to treat diseases of the entire vascular system. Intraoperative hemostasis, precise vision of intricate vascular anatomy, restenosis, implant-associated infection, and inadequate endothelium regeneration are among the enduring difficulties that continue to limit vascular surgery. Advanced biomaterial techniques are required since conventional materials and imaging technologies often fail to meet these multifactorial limitations. By enabling synthetic nanomaterials with programmable physicochemical qualities that directly interact with biological systems at the molecular and cellular levels, recent developments in nanotechnology have sparked a paradigm shift in vascular surgery. The limitations of conventional contrast media are addressed by nanostructured contrast agents, such as superparamagnetic iron oxide nanoparticles, which significantly improve vascular imaging resolution and diagnostic sensitivity across magnetic resonance modalities. Biodegradable polymers and surface-engineered coatings are examples of controlled-release nanocarriers that enable targeted administration of antiproliferative and antithrombotic drugs, lowering systemic toxicity and minimizing restenosis. In addition to providing long-lasting antibacterial action to avoid surgical site infections, nano-engineered surface topographies and coatings on grafts and stents accelerate endothelialization and reduce thrombogenicity. Additionally, some nanomaterials’ inherent catalytic and redox modulation properties aid in better wound healing and inflammation resolution. With a focus on mechanistic insights into hemostasis, imaging enhancement, drug transport, implant integration, and regenerative processes, this review thoroughly summarizes recent preclinical and translational studies on the use of nanomaterials in vascular surgery. Furthermore, we outline the present obstacles to clinical translation, such as biocompatibility, long-term safety, and manufacturing scalability, and suggest future paths for incorporating nano-enabled techniques into evidence-based surgical practice.

## 1 Introduction

Vascular surgeries are carried out to restore the blood flow in peripheral artery disease, aneurysms, and thrombotic disorders (Calligaro et al., 2008; Nott, 2022). Vascular surgeries are applied when advanced vascular dysfunctions, including molecular, cellular, or hemodynamic abnormalities of blood vessels, as well as loss of structure occurs (Wang Z. et al., 2021; Zhang et al., 2024). For instance, atherosclerotic plaque formation (narrows the vascular lumen and restricts blood flow) (Holmstedt et al., 2013; Xie et al., 2023; Zhang et al., 2024) as well as myocardial infarction and cerebral ischemia (major events in acute vascular occlusion) require urgent surgical or endovascular management (Zhang et al., 2024). In addition, tumor angiogenesis and inflammation result in structurally and functionally unstable vasculature, which can complicate surgical access and repair (Chen and Cai, 2014; Dvorak, 2015). Moreover, surgical techniques, even after advancement, could lead to various complications such as neointimal hyperplasia, thrombosis, and graft failure (Gaudino et al., 2017), due to inadequate drug delivery, delayed endothelialization, and limited biocompatibility of conventional implants (Bai et al., 2022; Melchiorri et al., 2015). Under these conditions, precise identification of vascular dysfunction is critical for selecting appropriate surgical or endovascular interventions (Zhang et al., 2024). Vascular implant failure is primarily driven by local biological responses that compromise long-term patency and function. Among these, intimal hyperplasia is a predominant cause of intermediate-term graft failure in both autologous and synthetic vascular grafts. Intimal hyperplasia results from endothelial injury and disturbed hemodynamics at anastomosis sites, leading to maladaptive remodeling that narrows the lumen and predisposes to secondary occlusion. Recent reviews highlight the role of compliance mismatch and flow disturbances in accelerating this process and limiting graft performance in small-diameter applications (Zizhou and Wang, 2022; Yao and Pohan, 2023). Early thrombotic occlusion also critically undermines implant patency. Thrombosis is initiated by endothelial disruption and biomaterial surface incompatibility, prompting platelet activation and fibrin deposition soon after implantation. Anticoagulant strategies and optimized surface hemocompatibility have been explored to mitigate this risk, as unresolved thrombus can lead to acute closure of the implant (Fang and Ellman, 2021). Prosthetic vascular graft infection represents another serious failure mode with high morbidity. Infection may arise from intraoperative contamination or biofilm formation on the implant surface, with common pathogens such as *Staphylococcus aureus* implicated in persistent infection and graft loss. Recent comprehensive overviews discuss the pathogenesis and clinical management of vascular graft infection (VGEI), emphasizing diagnostic and antimicrobial strategies (Costa and Andreucci, 2023).

Recent advances in nanotechnology have led to its use in various medical fields, such as cancer therapy and drug delivery (Klusman and Martin, 2023; Barcena and Perez, 2024; Wang and Xu, 2024; Zeng and Zhang, 2025). Nanomaterials have been progressively incorporated into the field of vascular surgery for imaging, hemostasis, drug delivery, and surface engineering of implants (Abaszadeh et al., 2023). The relevance of nanotechnology to clinics comes from its ability to increase the sensitivity, accuracy, and duration of results (Ma et al., 2024). Nanomaterials can be modulated in size, surface chemistry, mechanical rigidity, and degradation rates, down to nano scales (Dagdag et al., 2024), that directly interact with vascular cells, blood components, and the extracellular matrix (ECM).

**FIGURE 1:**
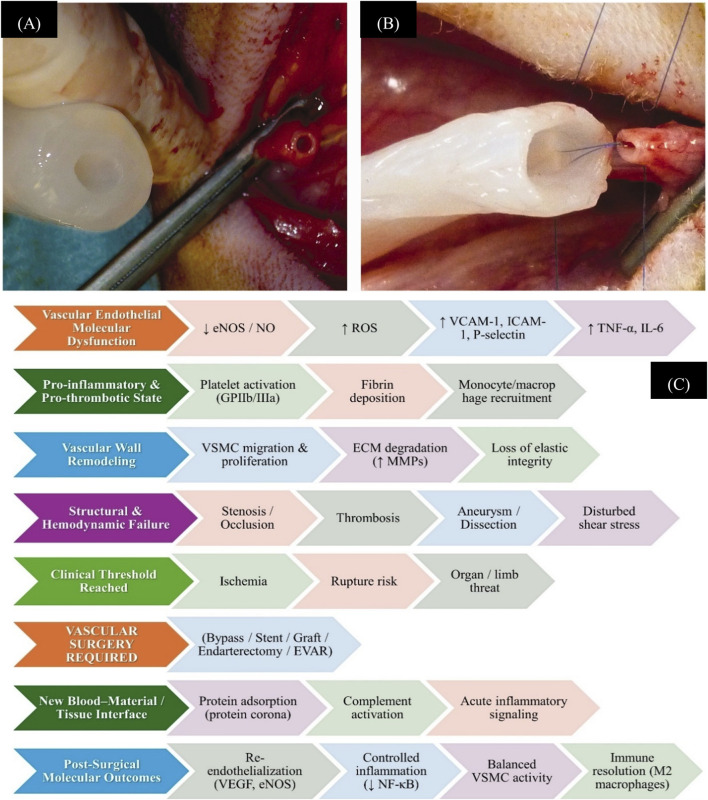
Implantation of thicker first-generation **(A)** and thinner second-generation **(B)** bacterial nanocellulose tubes during surgery. Retrieved from (Fodor et al., 2021). **(C)** Molecular progression of vascular dysfunction leading to surgical necessity and post-surgical healing. The cascade depicts key stages from endothelial dysfunction (↓eNOS/NO, ↑ROS, ↑VCAM-1/ICAM-1/P-selectin, ↑TNF-α/IL-6) to pro-inflammatory and pro-thrombotic states, vascular wall remodeling, and structural/hemodynamic failure. Nanoparticle interventions can target multiple points in this sequence: HDL-mimetic nanoparticles act at early endothelial dysfunction and inflammation to reduce ROS and cytokine signaling; cell-membrane-coated nanoparticles and hybrid nanocarriers modulate platelet activation, fibrin deposition, and immune cell recruitment; and nanoparticle-based coatings or drug delivery systems on vascular grafts/stents influence post-surgical outcomes by promoting re-endothelialization, controlling inflammation (↓NF-κB), and balancing VSMC activity, ultimately facilitating immune resolution (M2 macrophage polarization).

For vascular repair, autologous vessels/grafts are utilized in arteries and veins in the upper or lower limbs. However, the limitations, such as amputation, prior harvest, vascular disease in autologous vessels, may result in donor site morbidity. Therefore, a synthetic vascular graft is needed as an alternative for vascular restoration. i.e., bacterial nanocellulose grafts (Figure 1A,B) (Fodor et al., 2021). The pre- and post-vascular surgery molecular modulations in vascular architecture in disease conditions are described further in Figure Legends Figure 1C. This review focuses on the critical analysis of physically supported nanomaterial systems, which have shown functional superiority over traditional methods in vascular surgery, specifically novel NP formulations, nano-engineered stents, grafts, and surface functionalization.

## 2 Nanomaterials based diagnostics and intra-operative tools in vascular surgeries

### 2.1 Imaging with nanoparticles

NP-based contrast agents improve vascular imaging by reducing signal-to-noise ratios, prolonging intravascular residence times, and permitting molecular targeting of vascular disease. Their utility arises from size-dependent magnetic, optical, and X-ray attenuation properties that permit functional imaging of inflammation, thrombosis, and endothelial injury beyond anatomical assessment alone (Zhang et al., 2024). Despite extensive preclinical development, only a limited subset of NP-based contrast agents has achieved routine clinical adoption (Miyasato et al., 2021). They have the ability to interact at supramolecular, molecular, and even at atomic level, which makes them the most suitable material to detect and characterize single molecules, i.e., DNA, RNA or proteins, with NP-based platforms (Larguinho et al., 2015; Mariappan, 2019). The higher surface area of NPs facilitates better conjugation with imaging probes, targeting ligands, and stabilizing coatings, thereby signal intensity and specificity is increased compared with conventional small-molecule contrast agents (Singh et al., 2024). Furthermore, NPs can selectively leverage altered endothelial permeability, surface receptor expression, and inflammatory vascular microenvironments present under surgical conditions (Estelrich et al., 2015). In addition, NPs have been thoroughly investigated as contrast agents in biomedical imaging, in addition to their therapeutic potential. Superparamagnetic iron oxide NPs (SPIONs), for instance, have been used as FDA-approved off-label contrast agents in magnetic resonance imaging (MRI) because of their superior biocompatibility profiles, great magnetic responsiveness, and *in vivo* durability (Miyasato et al., 2021). NPs can create local magnetic field inhomogeneities that can accelerate proton relaxation. This relation creates a higher contrast and improves visibility of vascular structures and diseased regions (Zhou et al., 2017). SPIONs improve MRI sensitivity by generating significant T2/T2* contrast and inbuilt macrophage-rich or damaged endothelium areas which allow neointimal hyperplasia identification soon after angioplasty or bypass grafting (Estelrich et al., 2015; Li et al., 2023). Furthermore, gold NPs are used as efficient CT contrast agents. Their high atomic number creates superior X-ray attenuation. Chemical modification of their surface enables them to target ligands. For example, RGD peptides upregulate and bind αvβ3 integrins during vascular remodeling, which assists surgeons to distinguish actively healing segments from early stenotic zones (Au et al., 2013). Although SPIONs and MRI contrast provide significant information on tissue and vessel characteristics, the clinical imaging landscape for vascular surgery is dominated by CT and fluoroscopy. CT angiography (CTA) and conventional fluoroscopy offer high spatial and temporal resolution, enabling rapid acquisition of high-resolution vascular maps and real-time guidance during interventional procedures. CTA and digital subtraction angiography remain frontline imaging tools due to these advantages, ease of integration into surgical workflows, and operator familiarity (Murphy and Aghayev, 2018; Kilbride and Narsinh, 2022; Rogers, Campbell-Washburn et al., 2023). MRI/contrast agents, including SPIONs, have limitations in spatial resolution and real-time guidance compared with CTA/fluoroscopy are often used for pre-procedural assessment, tissue characterization, or follow-up rather than intra-operative navigation (Murphy and Aghayev, 2018).

In addition, quantum dots are used for experimental vascular imaging. They provide great photostability and tunable fluorescence. Quantum dots and antibody conjugates against collagen IV, intercellular adhesion molecule 1 (ICAM-1), or vascular cell adhesion molecule 1 (VCAM-1) are used to identify inflammatory or unstable vascular surfaces that might need surgical reinforcement. Nevertheless, their preclinical and *ex vivo* settings usage is prohibited due to heavy metal toxicity and long-term retention (Jayagopal et al., 2007). Several studies have demonstrated the ability of NPs engineered to bind fibrin (Liu et al., 2025; Sun et al., 2020), oxidized LDL (Chnari et al., 2006; Chunta et al., 2025), or collagen (Mozaffari et al., 2025) to identify microthrombi, plaque activation, or aneurysmal degeneration. Such molecular imaging shows up pathological segments even before morphological changes become angiographically apparent, enhancing surgical planning and allowing refined intra-operative navigation (Alam et al., 2018).

### 2.2 Radiopaque nanoparticles as device-embedded contrast agents for absorbable vascular devices

Beyond their role as intravascular contrast agents, nanoparticles have increasingly been integrated directly into vascular implants to enable intrinsic radiopacity and longitudinal device visualization. This strategy is particularly relevant for bioresorbable vascular devices, where loss of visibility following implantation remains a major clinical limitation. Conventional polymeric absorbable devices are radiolucent, complicating deployment accuracy, post-operative monitoring, and assessment of degradation, migration, or fracture. Radiopaque nanoparticles (including gold, tantalum, bismuth, tungsten, and iodine-containing nanocomposites) have emerged as effective solutions for imparting X-ray and CT visibility without compromising mechanical integrity or biodegradation kinetics (Emonde and Eggers, 2024). When embedded within polymer matrices such as poly-L-lactic acid (PLLA), polycaprolactone (PCL), or polydioxanone, these nanoparticles create uniform, stable contrast signatures that persist throughout the functional lifetime of the device and gradually diminish as resorption progresses (Tian and Lee, 2017; Pawelec and Hix, 2024). Recent studies demonstrate that gold nanoparticle–doped biodegradable scaffolds provide high-resolution fluoroscopic and CT visualization while maintaining cytocompatibility and controlled degradation behavior (Tian and Lee, 2017). Gold’s high atomic number enables strong X-ray attenuation at relatively low filler concentrations, minimizing adverse effects on polymer mechanics or inflammatory response. Similar outcomes have been reported for tantalum oxide and bismuth oxide nanoparticles, which offer excellent radiopacity and chemical stability, making them particularly attractive for long-term implant monitoring (Emonde and Eggers, 2024; Pawelec and Hix, 2024). This approach has shown strong translational relevance in absorbable inferior vena cava (IVC) filters, where real-time fluoroscopic visibility is critical for safe deployment and retrieval assessment. Nanoparticle-enabled radiopaque IVC filters allow clinicians to track device expansion, strut integrity, tilt, migration, and gradual bioresorption using standard clinical imaging systems, without the need for secondary contrast injections (Tian and Lee, 2017). Comparable strategies have been applied to vascular grafts and bioresorbable stents, enabling non-invasive monitoring of graft patency, deformation, and structural remodeling over time (Ding and Fu, 2022). Importantly, device-embedded radiopaque nanoparticles support longitudinal, contrast-agent-free imaging, reducing patient exposure to nephrotoxic iodinated contrast media and enabling repeated follow-up scans (Emonde and Eggers, 2024). This is particularly advantageous in patients requiring long-term surveillance after complex vascular reconstructions. Moreover, nanoparticle distribution within the implant can be engineered to generate spatial contrast patterns, allowing visualization of localized degradation or mechanical stress concentration (Pawelec and Hix, 2024).

### 2.3 Nanomaterials for hemostasis and surgical assistance

Uncontrolled bleeding remains a major cause of preventable mortality in trauma and vascular surgeries, underscoring the critical need for rapid and effective hemostatic control (Jiao et al., 2023). Traditional hemostatic agents often fall short in the high-pressure, dynamic environment of arterial flow (Guo et al., 2023). Current coagulation cascade and adhesive agents for hemostasis include adhesives, bandages, dressings, sealants, hemostatic powder, glue, and tissue adhesives. Widely used options as adhesives, such as fibrin glue and cyanoacrylates suffer from limited adhesion or biosafety concerns (Jiao et al., 2023). In addition, external hemostats such as kaolin- and zeolite-based products and biopolymer-based materials, including chitosan, gelatin, alginate, and collagen, have recently been developed to improve rapid bleeding control in clinical and surgical settings (Hickman et al., 2018).

**FIGURE 2:**
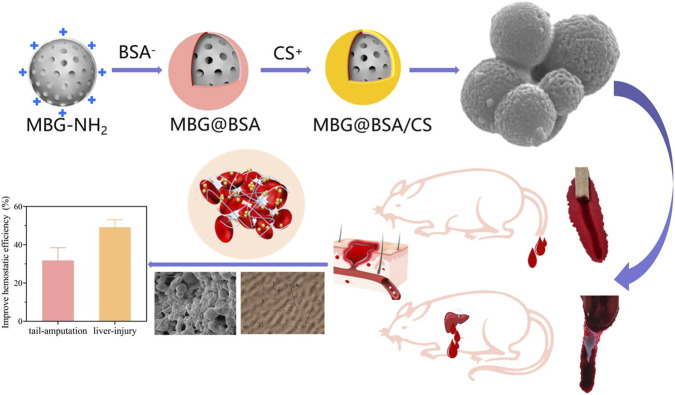
A schematic workflow and effectiveness of layer-by-layer MBG@BSA/CS applied by Zheng et al.(2022). BSA (bovine serum albumin) and CS (chitosan). MBG (mesoporous bioactive glass). Copyright © 2022 The Author(s). Published by Elsevier Ltd.

Nanomaterials play a major role in intra-operative hemostasis, which is crucial for vascular procedures where precise blood management is necessary to avoid ischemic consequences (Du et al., 2023). Nanomaterials are arising as a new generation of hemostats that biomimic and interact effectively, offering superior control and efficacy (Du et al., 2023). For example, chitin driven chitosan biopolymer, which when engineered into NPs, results in high density cationic surface that can bind to anionic phospholipids on the membranes of erythrocytes and platelets (Yadav et al., 2024). This reaction triggers the aggregation of blood cells and the activation of platelets. This results in the formation of a stable adhesive plug at the site of vascular injury, with concentrated coagulating factors. This process is very effective in diffusing bleeding in the microvasculature (Gheorghiță et al., 2023). Zheng et al. observed activation of physiological coagulation pathways, as well as significant surface and internal hemostasis, in SD rats. They applied encapsulated bovine serum albumin (BSA) and chitosan (CS) in a mesoporous bioactive glass (MBG) nanoparticle (MBG@BSA/CS) (Du et al., 2023; Zheng et al., 2022). Their effective hemostasis scheme is given in Figure 2.

**FIGURE 3:**
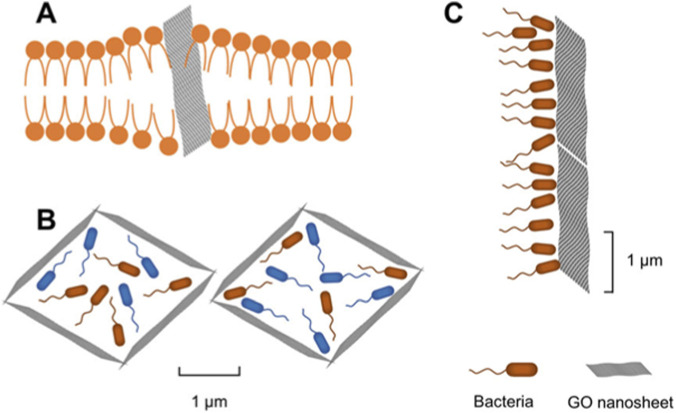
GO nanosheets display antibacterial activity via **(A)** integration in the cell membrane **(B)** capturing bacteria to absorb and encapsulate bacteria, and **(C)** physically inhibiting bacteria. Retrieved from (Li S. et al., 2025).

Further, graphene oxide (GO), a carbon-based nanomaterial, also has hemostatic properties (Díez-Pascual, 2021). The effectiveness of GO nano-sheets is dependent on a large surface area containing concentrated oxygen molecules attached to functional groups (Wu et al., 2022). The surface charge of GO NPs induces platelet pathway, oxidative stress, and adsorption of coagulation proteins (Li S. et al., 2025). In addition, GO nanocomposites as elastic meshes or injectables are generated for irregular wound topologies, where they maintain a strong mechanical barrier at extreme arterial pressure. Moreover, peptide nanofibers can assemble automatically, which is a biomimetic property of NPs. This auto-assembly process instantly produces a faux ECM with a sealant effect on tissue matrix (Hellmund and Koksch, 2019), and form a non-toxic mechanical seal which does not affect the post healing process (Danker et al., 2021). In addition, the antibacterial activity of GO-nanosheets is via cell membrane integration, trapping bacteria in a barrier followed by their absorption and creating a physical barrier for bacteria to cross, as shown in Figure 3 (Li S. et al., 2025).

Nanomaterials, besides hemostasis, when used in coatings, also have safety or antimicrobial properties for surgical instruments (Du et al., 2023). Antimicrobial coatings are directly applied to surgical instruments, sutures, and implant materials themselves (Sahoo et al., 2022). For example, silver NPs (AgNPs) are rich in antimicrobial properties. This antimicrobial activity is dependent on its silver (Ag^+^) ion, which targets the bacterial cell membranes, respiratory enzyme complex functions, and DNA, inhibiting the growth of both planktonic cultures and biofilm-forming microbes (Wang L. et al., 2021). However, their cytotoxic effects are to be countered by embedding them into a biocompatible polymer, such that the ion release rate can be modulated as per need. The titanium dioxide (TiO_2_) nanotubes lined on the surfaces of implants cause a photoactive antimicrobial effect. They produce reactive oxygen species (ROS) under surgical lighting conditions, which can kill bacteria by oxidizing bacterial cell components (Das et al., 2021; Junkar et al., 2016; Manisekaran et al., 2025). A broader-spectrum anti-bacterial and antifungal effect is also produced by copper oxide nanofilm interlayers, which results in rapid endothelial cell growth and healing while limiting joint infection (Manisekaran et al., 2025). Such nanoengineered biomaterials can minimize blood loss, increase surgical site visibility, and reduce ischemic complications. Whereas, their antimicrobial activity lowers the surgical site as well as prosthetic infections (Jaffer et al., 2007; Luo et al., 2024; Nahrendorf et al., 2009). Nanomaterial’s dual nature increases safety and success rates in complex vascular reconstructions, which may increase patient outcomes and reduce the cost of hospitalization (Luo et al., 2024).

### 2.4 Intraoperative vascular visualization and flow assessment enhanced by nanotechnology

Real-time visualization of endothelial rupture, thrombus formation, and inflammatory activity is important in vascular biology. The traditional imaging techniques provide only a limited insight into blood flow and patency, mostly failing to depict the molecular and biomechanical precursors of graft failure or thrombosis (Jaffer et al., 2007; Matsui et al., 2009). Nanomaterials made this intra-operative visualization of vasculature possible. The real-time visualization can facilitate identifying diseased arterial segments at the molecular level that apparently may appear intact. The targeted nanoprobes are functionalized against fibrin, collagen exposure, or adhesion molecules to improve intraoperative visualization and decision-making (Matsui et al., 2009; Shaw, 2009; Tange et al., 2024). The first innovation consists of targeted fluorescent nanoprobes (Falk et al., 1995). Precisely, molecular targeting can significantly advance cardiovascular molecular imaging, and a timely intervention can be applied long before structural deterioration becomes irreversible (Jaffer et al., 2007; Matsui et al., 2009).

In near-infrared fluorescence, innovation is made in extending the utility of basic perfusions. For example, indocyanine green encapsulated dye engineered with nanocarriers overcomes traditional limitations in rapid photobleaching and vascular washout (Zhang et al., 2024). These nano formulations behave as stabilized, high-fidelity tracers. In real time, due to their prolonged circulation and enhanced signal, free flap transfers or distal bypasses can be assessed. Surgeons cannot only observe initial blush but also observe sustained capillary-level uptake. Thereby, marginal tissues at risk for necrosis, that would otherwise appear normal to conventional inspection, can be identified. This measurement based on estimation eventually can reduce re-operation rates (Alam et al., 2018; Matsui et al., 2009).

The molecularly targeting nanoprobes are designed for intraoperative usage. Conventional imaging identifies a narrowed segment, while a probe targeting fibrin can illuminate a nascent angiographically occult thrombus at a suture line (Liu et al., 2025; Sun et al., 2020). Likewise, NPs can delineate regions of endothelial denudation or pronounced inflammation adjacent to a stent or graft (Jaffer et al., 2007; Mozaffari et al., 2025).

### 2.5 Theranostic nanoparticles for integrated vascular imaging and localized therapy

Theranostic nanoparticles integrate diagnostic imaging and localized therapy, enabling real-time visualization and targeted treatment of vascular pathologies such as inflammation and atherosclerosis. These platforms frequently utilize imaging cores like superparamagnetic iron oxide nanoparticles (SPIONs) for MRI or other contrast agents, combined with drug-loaded polymeric or lipid shells for therapeutic delivery, allowing detection of inflamed endothelium and plaques alongside therapeutic intervention (MacRitchie, Di Francesco et al., 2021; Wang and He, 2024). Functionalization with targeting ligands such as VCAM-1 peptides enhances selective accumulation at sites of vascular injury, enabling precise therapeutic delivery and reducing complications such as restenosis or plaque rupture (Li and Wu, 2022). Additionally, stimulus-responsive designs have been explored that release drugs in response to local cues (e.g., oxidative stress or acidic microenvironments), maintaining imaging functionality while optimizing spatial and temporal drug delivery for guided vascular interventions and postoperative monitoring (Xu and Li, 2022).

### 2.6 Nanobiosensors for rapid detection of cardiac and vascular biomarkers

Nanobiosensors leverage nanomaterials and advanced transduction mechanisms to detect biomarkers with high sensitivity and rapid response. Electrochemical nanobiosensors, optical nanobiosensors, and plasmonic platforms are widely investigated for cardiac biomarker detection. Electrochemical nanobiosensors have been widely developed for cardiac troponin I (cTnI) detection due to their rapid response and low detection limits, making them ideal for early acute myocardial infarction (AMI) diagnosis. Materials such as carbon-based nanostructures, metal matrix composites, metal-organic frameworks (MOFs), and conductive polymers enhance signal transduction and lower limits of detection, facilitating high-precision, rapid quantification of cTnI in point-of-care settings (Zhang and Zhu, 2025). Optical nanobiosensors also provide real-time, label-free detection using localized surface plasmon resonance (LSPR), surface-enhanced Raman scattering (SERS), and fluorescence methods. These platforms can detect low pg/mL concentrations of cardiac biomarkers and support multiplexing for simultaneous measurement of multiple analytes (e.g., cTnI, BNP, CRP) (Vairaperumal and Huang, 2023; Davronova, Siradjitdinova et al., 2025). Furthermore, nanobiosensors targeting non-protein biomarkers such as microRNAs offer additional insights into disease progression and severity. Electrochemical miRNA sensors functionalized with nanostructured electrodes demonstrate rapid and sensitive detection of disease-related miRNAs, expanding the diagnostic utility beyond traditional protein biomarkers (HalvaeiKhanekahdani and Wu, 2025).

## 3 Nanomaterials for drug delivery in vascular surgical management

### 3.1 Nanomedicine based carriers to stop restenosis

Restenosis is the narrowing of a blood vessel after it has been expanded by surgery, such as angioplasty. It is frequently caused by scar tissue (neointimal hyperplasia) growing within a stent or artery. NP-based antiproliferative agents while minimizing systemic exposure. For example, paclitaxel stabilizes microtubules and arrests VSMCs in the G2/M phase of cell division. Sirolimus inhibits mTOR signaling and blocks the G1 to S phase transition in cell division. PLGA encapsulation provides release kinetics aligned with the temporal dynamics of neointimal formation (Bai et al., 2022; Iyer et al., 2019). Besides this passive release, active targeting can also be achieved by functionalizing the surfaces of NPs with ligands that bind to molecular markers on activated endothelium (E-selectin). This results in concentrating this antiproliferative payload at the precise cellular interface where pathology initiates (Choi et al., 2022; Matoba and Egashira, 2014).

### 3.2 Targeted nanomedicine for inflammation and thrombosis

Thrombosis and inflammation in vascular injury are due to either surgical trauma or device implantation. Both processes initiate a dangerous cascade where one process fuels the other. Traditional antiplatelet or anti-inflammatory drugs act globally. Therefore, they cannot stop local clotting and lead to systemic bleeding. Targeted nanomedicine precisely recalibrate this balance by delivering therapeutic cargo just to the activated endothelium and circulating diseased cells (Choi et al., 2022; Matoba and Egashira, 2014). Different molecular logic underlies in nanomedicine targeted approach. For example, endothelium expresses surface adhesion molecules like P-selectin and becomes sticky, These p-selectins (P-selectin glycoprotein ligand-1 (PSGL-1)) can be targeted with NPs, precisely surgical damage can be located (Choi et al., 2022). After locating the damage with PSGL-1, payloads of anti-inflammatory agents (e.g., inhibitors of the NF-κB pathway) can be released to stop the inflammatory cytokines like IL-6 and TNF-α. Simultaneously, thrombosis threat is also stopped with specificity. For example, NPs with fibrin-specific peptides or monoclonal antibodies against GPIIb/IIIa have high binding affinity and to develop thrombi (Liu et al., 2025; Sun et al., 2020). Therefore, direct thrombin inhibitors or platelet-disaggregating agents concentrate at their action at the clotting site without disturbing systemic circulation (Matoba and Egashira, 2014).

### 3.3 Nano-engineered vascular implants and bio-functional surfaces

Vascular implants that are made small can give mechanical support and have surfaces that are made to work with the body. These surfaces help with blood clotting and inflammation. Also help the inside of the implant feel like a natural part of the body. The implants have holes and tubes that increase surface area, which allows for controlled release of medicine. This special design also helps the right kind of cells stick to the implant and grow. The implant is made in layers, which lets it release different kinds of medicine at different times. Ultimately, the inflammation is reduced, and cell growth is inhibited. For its proper working, implant surfaces are designed as that it attracts required cells. The drug is loaded into tiny holes and tubes in vascular implants to help heal. In bio-degradable implant systems, synchronized mechanical support with vascular remodeling of nanocomposites modulates radial strength and degradation kinetics.

Basic mechanical scaffolded drug-eluting devices are advanced to bio-responsive and active platforms. Although conventional drug-eluting stents (DES) have made significant advancements. However, their polymer coatings occasionally could cause uneven drug distribution or localized inflammation. Nanotechnology turns passive elution into a planned therapeutic effect, as it proposes device-tissue interface modification at a molecular level (Iyer et al., 2019). Stent struts with nano-porous and nano-tubular surfaces produced by anodization or etching provide a significantly larger surface area, which facilitates controlling the temporal and spatial dynamics of drug release. Hence, an initial burst release and subsequent troughs, that can undermine efficacy, are prevented (Bai et al., 2022).

Nanotechnology allows sequential and multifunctional delivery that goes beyond kinetics. Drug-loaded polymers can be deposited in alternating layers using layer-by-layer nano-assembly. This can be made to release an anti-inflammatory medication (like dexamethasone) to reduce surgical trauma during the first week, and then to continuously elute an anti-proliferative medication (like sirolimus) to fight hyperplasia. (Iyer et al., 2019). Additionally, pro-healing molecules can be combined with antiproliferative medications in nano-coatings. For example, peptides like REDV (Arg-Glu-Asp-Val) can specifically attract circulating endothelial progenitor cells to speed up re-endothelialization, while co-immobilizing CD47 mimetic peptides can send a “don’t-eat-me” signal to macrophages, reducing the foreign body response. (Melchiorri et al., 2015). Drug-eluting balloons (DEBs) are a powerful application of this principle. In this case, the stabilization of lipophilic medications, such as paclitaxel, on the balloon surface during transit and their effective transfer to the vessel wall upon inflation are made possible by NPs. Particle adhesion is increased, and a more homogeneous coating is produced when the medication is formulated as nanocrystals or loaded into lipid-based NPs. These NPs improve drug uptake by the arterial tissue upon balloon-wall contact, offering a powerful single-dose treatment without the need for a permanent stent, a critical benefit in complex lesions or infected fields. (Iyer et al., 2019). The creation of completely bioresorbable vascular scaffolds (BVS) with nano-engineered degradation profiles is the ultimate frontier. Ceramic NPs (i.e., hydroxyapatite) along with a polymer matrix are made to preserve the scaffold’s mechanical strength and radial force, while controlling degradation. The released NPs can be engineered, before the device harmlessly dissolves, for the healing process, as eroded polymer produces a final therapeutic act (Melchiorri et al., 2015).

Advanced nano-based devices are being advanced with complex bio-interfaces. Currently, next-generation biodegradable stents are being developed with nano-porous metallic alloys (e.g., magnesium-based) for intended controlled corrosion, mechanical resilience, and localized therapeutic ion release, as well as optimizing the NP carrier itself for drug-eluting balloons, such as employing lipid-core or polymer-lipid hybrid NPs to improve paclitaxel’s pharmacokinetic profile (Islam et al., 2024; Iyer et al., 2019; Jacob et al., 2025; Jiang et al., 2017). Nano implants that first support a healing artery and then withdraw, leaving only healthy tissue behind, are being introduced. Nano-engineering rethinks the stent as a dynamic, three-dimensional interface designed for effective biological repair (Iyer et al., 2019; Jiang et al., 2017). The nano-pores in implants serve as reservoirs, greatly increasing the capacity for drug loading and allowing for controlled, sustained release that aligns with the biological timeline of wound healing (Bai et al., 2022). By using layer-by-layer nano-assembly, we can coat a stent with alternating thin layers of different polymers and drugs (Iyer et al., 2019). The timing, similar to a planned therapeutic dialogue with the artery wall, marks a major improvement over first-generation drug-eluting stents (Bai et al., 2022). The imaging-specific nanomaterial platforms, including SPIONs, gold nanoparticles, and quantum dots, and their corresponding diagnostic advantages are comparatively summarized in Table 1.

**TABLE 1:** Nanomaterials and their mechanistic roles in vascular surgery.

Nanomaterial	Composition	Application	Mechanism of action	Surgical benefit	Level of evidence	Stage of development	Examples	References
SPIONs	Fe_3_O_4_/Fe_2_O_3_	MRI/MRA imaging	T_2_/T_2_* contrast via magnetic field inhomogeneity; macrophage uptake	Intraoperative visualization, inflammation mapping	Human observational studies; animal models	Approved (off-label clinical use)	Ferumoxytol	Shen et al. (2024)
Gold NPs	Au	CT angiography	High X-ray attenuation; ligand targeting (RGD-αvβ3)	Precise lesion localization	Small-animal studies	Preclinical	CT vascular contrast	Au et al. (2013)
Quantum dots	CdSe/ZnS	Experimental vascular imaging	Stable fluorescence, molecular targeting	Preclinical molecular mapping	*In vitro* and small-animal studies	Preclinical	ICAM-1–QD probes	Jayagopal et al. (2007)
Chitosan NPs	Deacetylated chitin	Hemostasis	Electrostatic platelet aggregation	Rapid bleeding control	Rodent and large-animal models	Preclinical	Surgical sealants	Du et al. (2023), Zheng et al. (2022)
Graphene Oxide	Carbon nanosheets	Hemostasis, antimicrobial coatings	Platelet activation, protein adsorption, ROS	Bleeding and infection control	*In vitro* and animal models	Preclinical	GO meshes	Díez-Pascual (2021), Li S. et al. (2025)
Silver NPs	Ag	Antimicrobial graft coatings	Ag ion release, enzyme inhibition	Infection prevention	Human clinical studies	Clinically approved/in use	Silver-coated grafts	Ricco and Assadian (2011)
TiO_2_ Nanotubes	Titanium dioxide	Implant surface coatings	Photo-induced ROS generation	Long-term antimicrobial protection	*In vitro* and animal studies	Preclinical–early translational	TiO_2_-NT stents	Junkar et al. (2016)
PLGA NPs	Biodegradable polymer	Drug-eluting stents/balloons	Controlled drug release (paclitaxel, sirolimus)	Reduced restenosis	Large-animal studies; human RCTs	Clinically approved	DES, DEB	Iyer et al. (2019), Sharma et al. (2025)
Magnesium nano-alloys	Mg-based metals	Bioresorbable stents	Controlled corrosion, ion release	Temporary support	Large-animal and early human studies	Early clinical (Phase I/II)	BVS	Meng et al. (2025)
Hydroxyapatite NPs	Ca_10_(PO_4_)_6_(OH)_2_	Scaffold reinforcement	Mechanical strength, bioactivity	Graft durability	*In vitro* and animal studies	Preclinical	Composite grafts	Muñoz (2018)
Cerium oxide NPs	CeO_2_	Wound healing	ROS scavenging	Reduced inflammation	*In vitro* and animal studies	Preclinical	Surgical dressings	Wu et al. (2025)

### 3.4 Classes of nanocarriers for vascular biomedical implants: Lipid, polymeric, inorganic, and bioinspired systems

Lipid-based, polymeric, inorganic, and hybrid nanocarriers constitute the foundational delivery platforms underpinning modern nano-engineered vascular implants. Each class offers distinct physicochemical advantages in terms of drug encapsulation, release kinetics, hemocompatibility, and interface integration with stents, grafts, and drug-eluting balloons.

Liposomes are among the most clinically mature nanocarriers and have been extensively explored for vascular implant functionalization due to their biocompatibility, amphiphilic architecture, and capacity to encapsulate both hydrophilic and hydrophobic therapeutics. When immobilized on stent surfaces or incorporated into polymer coatings, liposomes enable localized and sustained elution of antiproliferative agents such as sirolimus and paclitaxel, thereby reducing neointimal hyperplasia while minimizing systemic exposure (Abbasi and Kouchak, 2022; Islam et al., 2024). Polymeric micelles, formed from amphiphilic block copolymers, offer superior solubilization of hydrophobic drugs and enhanced arterial wall penetration. PEG-based micellar coatings on drug-eluting balloons and stents have demonstrated improved drug transfer efficiency and uniform vessel wall deposition compared to crystalline drug coatings (Islam et al., 2024; Cho and Rasoulianboroujeni, 2025).

PLGA and PEG-PLGA nanoparticles are among the most extensively studied biodegradable polymeric systems for vascular drug delivery due to their tunable degradation and sustained release profiles, with applications demonstrated in intravascular drug delivery and cardiovascular disease contexts (Yang and Xue, 2022; Naskar et al., 2024; Yang and Zeng, 2024). Dendrimers, with their highly branched, monodisperse architectures and multivalent surface functionalities, facilitate high drug loading and ligand conjugation for advanced delivery applications, including nitric oxide release, gene transfection, and anti-thrombotic approaches; however, their long-term safety profiles continue to be actively investigated (Sun and Slomberg, 2012; Palmerston Mendes and Pan, 2017; Akhtar and Babiker, 2024).

Inorganic nanoparticles have been demonstrated to enhance both diagnostic imaging and functional performance when incorporated into vascular implants. For example, gold nanoparticles are effective contrast agents for X-ray/CT imaging and can accumulate at sites of vascular inflammation in experimental models, enabling non-invasive visualization of diseased arteries (Kosuge and Nakamura, 2022). Similarly, iron oxide nanoparticles provide magnetic resonance contrast enhancement and act as theranostic vectors with potential immunomodulatory effects relevant to macrophage-mediated inflammatory processes in vascular tissues (Ghazi and Ibrahim, 2025). Mesoporous silica nanoparticles offer a tunable high-surface-area platform for multi-drug loading and controlled release, although their long-term biodegradation and safety profiles remain active areas of investigation (Xu and Li, 2023).

Hybrid and bioinspired nanocarriers represent a rapidly advancing frontier in vascular nanomedicine. HDL-mimetic nanoparticles have been engineered to mimic endogenous HDL structure and function, facilitating interaction with macrophage receptors, promoting cholesterol efflux, and exerting anti-inflammatory effects at atherosclerotic and injured vascular sites, while offering high biocompatibility and low immunogenicity (Rani and Marsche, 2023; Zhen and Li, 2024). Cell-membrane-coated nanoparticles, derived from platelet, erythrocyte, or immune cell membranes, provide biomimetic cloaking that enhances immune evasion, prolongs circulation, and improves targeted delivery to inflamed endothelium and thrombotic regions, as detailed in recent cardiovascular reviews (Liu and Wang, 2025; Zhao and Chen, 2025).

## 4 Nanostructured vascular grafts

For vascular surgery, a major challenge is to search for a functional, durable, biocompatible, and small (<6 mm) diameter vascular graft. Traditional materials like Dacron and ePTFE are lifesaving, however, they are inert tubes that cannot endothelialize, which results in acute thrombosis, and their poor mechanical fit can lead to intimal hyperplasia, graft blockage, or aneurysmal dilation over time (Hernandez-Sanchez et al., 2024; Li Q. et al., 2025). These limitations in materials are handled in nanostructured grafts by chemical modification, mimicking native ECM and with increased healing potential (Haririan et al., 2025). For nanostructured grafts electrospinning method is extensively used due to its versatility and cost-effectiveness (Zulkifli et al., 2023). Electrospinning produces a non-woven mesh of polymer fibers with diameters in the nanometer to micrometer range (Naeim et al., 2025). The grafts produced through electrospinning with nano-topography facilitate, signaling and attachment of endothelial and vascular smooth muscle cells (VSMCs). Consequently, a continuous, functional endothelium, which acts as the body’s ideal natural antithrombotic surface, is produced. Additionally, the interconnected porosity of the electrospun matrix also facilitates nutrient and waste exchange, which reduces blood leakage (Melchiorri et al., 2015; Xie et al., 2023).

Furthermore, nanomaterials like carbon nanotubes, graphene oxide, or nanocrystalline ceramics (such as hydroxyapatite) supported grafts have higher tensile strength, suture retention strength, and flexibility (Jiang et al., 2017) which are vital to withstand arterial pressures, particularly for long-term implants. Moreover, magnetic NPs incorporated graft are further advantageous as they can precisely allow graft placement at the needed site. It can also facilitate a localized heat treatment, if needed, while applying an external magnetic field (Materón et al., 2021). Their nano-functionalized surfaces can also turn a passive scaffold into an active biological interface. For example, Melchiorri et al. (2015) suggested two strategies 1) to attach cell-adhesive peptides (like RGD), and 2) pre-seeding with endothelial cells. Both mechanisms can enhance endothelialization of the nano-grafts (Melchiorri et al., 2015).

In addition, the surface can also be modified with covalent attachments like heparin and vascular endothelial growth factors (VEGF). Heparin creates a strong, localized surface that resists blood clotting (Tsai et al., 2001). Whereas, VEGF attachment can actively attract EPCs from the blood to populate the graft area (or *in situ* endothelialization) in coronary artery bypass grafts (Snyder, 2015). These multi-faceted nano-structured graft designs not only aim to create host body accepted grafts but also facilitate them to make these grafts as the body’s own functional part of the circulatory system (Das et al., 2024).

First-generation BVS such as the Absorb scaffold (Abbott Vascular) were hindered by a combination of mechanical and biological limitations. These devices required relatively thick polymeric struts (≥150 µm) to achieve sufficient radial strength, leading to altered local flow dynamics, increased turbulence, and delayed endothelialization compared with metallic drug-eluting stents (DES). Thick struts protruding into the lumen disturb wall shear stress patterns, promoting intimal hyperplasia and thrombosis risk (Sakamoto and Jinnouchi, 2018; Toong and Ng, 2022; Jeong and Jeong, 2023). Nanocomposite and nanostructured materials enhance mechanical properties by enabling higher modulus and strength at lower thicknesses. For example, polymer nanocomposites reinforced with nanocellulose, carbon nanotubes (CNTs), or silica nanoparticles show improved tensile and compressive properties that allow thinner strut designs without sacrificing radial support (Toong and Ng, 2020; Hashemzadeh and Bina, 2024; Kumar and Kumar, 2025).

## 5 Nanomaterials for post-operative healing and tissue regeneration

### 5.1 Endothelial regeneration

The fate and effectiveness of any vascular intervention are dependent on its ability to regenerate a functional endothelium monolayer (Evans et al., 2021). Vascular interventions include endarterectomy, bypass graft, and stent implantation. This one layer of cells is thromboresistant, anti-inflammatory, and a permeability-regulating interface, as well as an ultimate vascular health protector. On the contrary, regeneration failure or delay of this layer includes thrombosis, intimal hyperplasia, and eventual blockage. Nanomaterials made vascular interventions help in the healing phase due to their pro-regenerative milieu that actively attracts, trains, and supports endothelial cells, going beyond passive scaffolding to bioactive instruction (Melchiorri et al., 2015). NPs can also modulate gene expressions and signals, either by directly delivering plasmid DNA or mRNA encoding for VEGF or other angiogenic factors to vessel wall cells. Consequently, host cells act as bioreactors which can produce the required proteins under physiological conditions (Zeng et al., 2025). Additionally, nanomaterials such as gold NPs, as signaling molecules, can alter intracellular pathways to induce endothelial cell migration and proliferation (Au et al., 2013; Liu et al., 2022). The basic idea behind nanotechnology for endothelium regeneration is collaborative healing (Aliyev et al., 2025; Roy et al., 2023).

### 5.2 Antimicrobial and anti-biofilm properties of nanoparticles

Microbes in biofilms, a slimy extracellular polymeric substance (EPS), formed by bacterial and fungal organisms, are challenging to kill by antibiotics and the immune system. Bacterial colonization can cause pathogenic infections in prosthetic grafts and stents (Su et al., 2025; Zhao et al., 2023). Infections that are associated with biofilms are a jeopardizing enemy for vascular implants (Mishra et al., 2024). The infections that arise from peri-operative contamination could result in high-risk explant surgery, septic emboli, and graft failure (Zabaglo et al., 2024). Therefore, nano-coatings offer a first-line defense with an active barrier that interferes throughout the lifecycle of biofilm formation (Das et al., 2021; Hellmund and Koksch, 2019). In addition, their physical and ion-mediated cytotoxicity inhibits antibiotic-resistant microbes. For example, silver NP (AgNP) coatings, due to their broad-spectrum activity against bacteria, fungi, and viruses, continue to be essential. Their antibacterial ability is due to prolonged release of silver ions (Ag^+^), which pierce microbial cell walls and attach to thiol groups in essential enzymes, causing cell death and metabolic disruption (Dube and Okuthe, 2025). This multi-target approach successfully stops the initial adhesion and proliferation phase that is essential for the formation of biofilms and makes it very difficult for bacteria to build resistance. (Hellmund and Koksch, 2019; Manisekaran et al., 2025). Further, photoactive nano-coatings such as titanium dioxide nanotubes (TiO_2_-NTs) provide an on-demand antibacterial activity against, for example, *S. aureus*, *P. aeruginosa*, *S. epidermidis*, and *E. coli*. TiO_2_ produces ROS, mostly hydroxyl radicals and superoxide anions, when exposed to ambient or surgical light, damaging bacterial cell walls, membranes, proteins, and nucleic acids (Roguska et al., 2012). This offers a real-life sterilizing impact, especially helpful in preventing infection during the implantation operation itself and throughout the implant’s lifetime (Junkar et al., 2016; Wang L. et al., 2021). Copper oxide nanofilms also offer strong antibacterial activity and possible pro-healing advantages. Like silver, they work by releasing biocidal ions (Cu^2+^) that interfere with metabolic processes and microbiological integrity. Copper ions (Cu^2+^) are also essential cofactors in human angiogenesis and ECM remodeling enzymes. Therefore, it can work for only defense but also in regenerative interfaces as a dual-function coating and enhance the endothelialization and tissue integration processes necessary for graft healing (Das et al., 2021; Manisekaran et al., 2025).

### 5.3 Nanomaterial effectiveness in immune modulation and wound healing

The vascular implant integration and surgical wound healing depend on the immune response’s coordination. Fibrosis, severe scarring, and poor tissue regeneration can result from an uncontrolled inflammatory phase. Advanced nano-dressings actively protect and heal the surgical wound site, while nanomaterials provide precise instruments to modify this immunological dialogue and change the milieu from pro-inflammatory to pro-healing (Du et al., 2023; Guo et al., 2023). NPs can be a tactic to specific immunomodulation. For example, small-molecule inhibitors of signaling pathways like NF-kB or anti-inflammatory cytokines like IL-10 have the ability to transport particular biological agents directly to the perivascular tissue surrounding a graft or stent (Choi et al., 2022). The nano-therapies can also reduce chronic inflammation, which may lead to graft encapsulation and failure by locally reducing the prolonged activation of macrophages and the production of pro-fibrotic signals. This promotes a regenerative rather than a fibrotic healing trajectory. Nanotechnology-enabled bioactive dressings are also beneficial for the care of exterior surgical wounds. Nanomaterials such as cerium oxide NPs can imitate antioxidant enzymes to scavenge excess ROS, reducing inflammation and boosting fibroblast activity at the surgical wound site (Jiang et al., 2017). Similarly, hydrogel dressings containing graphene oxide derivatives can offer a conductive scaffold with modest antibacterial activity that promotes cell migration and proliferation (Li S. et al., 2025). These cutting-edge dressings lower the risk of infection and dehiscence by actively reducing inflammation, maintaining a moist, protective environment, and speeding up the closure and strength of the surgical wound. By promoting a balanced immune response and the best possible wound healing, nanotechnology therefore expands its function from the intravascular area to the whole surgical domain (Guo et al., 2023; Li S. et al., 2025).

The collective summary of the nanomaterials which play a pivotal mechanistic role in vascular surgeries is given in Error! Reference source not found.

## 6 Safety, toxicity, and regulatory perspectives

Prior clinical and/or vascular surgery application of any nanomaterial, its safety profiling is essential (Naskar et al., 2024). The success of NPs is dependent on their surface reactivity, capacity to cross biological barriers, and sustained circulation, which also determine their biocompatibility, long-term biodistribution, and possible elimination (Khan and Hossain, 2022). A strong regulatory framework should be adopted for not only patient safety but also for translation of nanomaterial from laboratory to clinical practice (Jiang et al., 2017; Miyasato et al., 2021). One main barrier to their translation into clinical practice is hemocompatibility. NPs interact with plasma proteins and form a protein corona. This protein corona results in hemolysis and activates the complement system and platelet pathways (de la Harpe et al., 2019). The interactions between NPs and blood/serum proteins are hydrophobic, surface charge (zeta potential) dependent. Positively charged surfaces are more likely to damage erythrocyte membranes, hence cytotoxic. Thus, thorough *in vitro* hemolysis and thrombogenicity tests are essential (Singh et al., 2024; Zhou et al., 2017). Further distribution and elimination of NPs within and from the body, respectively, are also very important. Both these properties are given by their size, shape, and surface chemistry of NPs, and that ultimately determines how quickly and efficiently the kidneys will eliminate them, and how the liver and spleen will store them. Several NPs, like quantum dots or gold particles, are non-biodegradable, and over time, they build up in organs, including the liver and spleen. Further, the toxicity of by-products of biodegradable systems, PLGA by-products, such as lactic and glycolic acid, and their safe metabolic clearance must be proven in preclinical trials (Li et al., 2023; Miyasato et al., 2021). Importantly, their long-term toxicity is most concerning, which may result in fibrosis, low-grade inflammation, or aberrant immune systems. Additionally, nanomaterials may also act as adjuvants and modulate immune responses. To address this concern, human trials are necessary to check genotoxicity and immunotoxicity (Singh et al., 2024). The regulations for nano-enabled medical devices and therapeutics are evolving in tandem with technological advances. FDA and the European Medicines Agency (EMA) emphasize a thorough physicochemical characterization of nanomaterials, including size distribution, surface charge, aggregation state, and purity, as foundational to clinical translation (Miyasato et al., 2021). NPs’ behavior in biological systems can vary greatly, depending on test settings. Standardized techniques are also under development to test toxicity, degradation profiling, and performance of nano-enabled cardiovascular implants. A variety of orthogonal tests and thorough animal studies are being introduced to provide a convincing safety dossier until such regulations are fully enforced (Kurul et al., 2025; Singh et al., 2025). Furthermore, a transparent, thorough safety evaluation is essential to the long-term integration of nanotechnology in vascular surgery.

## 7 Clinical translation and current applications in vascular surgeries

Several nano-engineered systems are in the translational pathway in vascular surgery (Islam et al., 2024; Kuznetsov et al., 2020; Naskar et al., 2024; Rickel et al., 2021), even though many breakthroughs are still in preclinical development. These developments have the capacity of targeted delivery, increased compatibility, and enhanced imaging, as witnessed in human patients (Kurul et al., 2025; Singh et al., 2025). For example, nano-engineered first-generation DES transformed into interventional cardiology. However, DES may cause occasional local vascular irritation due to their polymer coverings. Nanoengineered stents/grafts offer nanoscale control over drug-release kinetics, which can be directly applied in therapeutic settings (Iyer et al., 2019; Jiang et al., 2017). Further, NP-loaded contrast agents are being utilized in clinical imaging procedures in the field of diagnostics. Ferumoxytol, an SPION, is frequently used as an off-label MRA contrast agent. It is FDA-approved for iron-deficient anemia. Additionally, macrophages absorb them, which enables a distinct detection of inflammatory activity within implanted grafts and artery walls (Estelrich et al., 2015; Miyasato et al., 2021). For surgical reconstruction, nano-functionalized vascular grafts are also advancing to clinical studies. These grafts have improved endothelial cell integration while reduced thrombogenicity effect. A serious complication in vascular surgery is prosthetic graft (silver or new composite nanomaterials) infections, which are reduced with antimicrobial nanocoating and are in clinical trials (Das et al., 2021; Liu et al., 2022; Melchiorri et al., 2015).

## 8 Challenges, knowledge gaps, and future perspectives

Even though nanomedicine for vascular surgery has advanced remarkably, there are still numerous scientific and translational obstacles in the way of widespread clinical integration (Kurul et al., 2025). To bridge this gap, laboratory settings, clinical nano products, manufacturing, biological interaction, and long-term safety of nanomaterials are suggested (Herdiana, 2025). Major difficulty is NP manufacturing is reproducibility and scalability (Fernandes et al., 2023). Further, multi-layered structures, precise ligand conjugates, or hybrid organic-inorganic compositions of nanomaterials need resilient good manufacturing practice (GMP) in their synthesis (Bi et al., 2025). The variation in batch to batch, in size distribution, surface charge, and drug loading efficiency may drastically affect their performance and toxicity in clinical practices (Ma et al., 2024). Such variations should be standardized with a scalable standard technique (Hachhach et al., 2025). Safe clinical translation of nanomaterials in vascular surgery requires compliance with ISO standards and nano-specific risk assessment due to their size-dependent properties (Sharifi and Mahmoud, 2022; Caputo and Favre, 2024). The ISO 10993 series guides biological evaluation of nano-enabled devices, including hemocompatibility and cytotoxicity, although conventional assays may overlook nano-specific effects such as protein corona formation and surface reactivity (Scimeca and Verron, 2022; Hajipour and Safavi‐Sohi, 2023). ISO/TR 13014 and ISO/TR 16197 provide frameworks for physicochemical characterization and toxicological screening, while ISO 22442 addresses safety concerns related to biodegradable or biologically derived nanocomposites (Sharifi and Mahmoud, 2022; El-Sayed and Youssef, 2023). Despite these standards, key challenges remain, including hemocompatibility risks from protein corona-mediated interactions, long-term nanoparticle accumulation and chronic toxicity, immune modulation, and manufacturing variability. These issues highlight the need for advanced testing strategies and strict quality-by-design approaches in nanomedicine.

The knowledge regarding dynamic nano-bio interactions needs to be extended such that a precise interacting nanomaterial is designed. Nanocoating create proteins covers called protein corona, which dramatically changes the NP’s effective identity in a biological system (Bashiri et al., 2023). It is the protein corona that cells and tissues recognize, not the surface of the artificial NPs. Its composition is intricate, fluctuating, and dependent on the patient’s particular physiological state as well as the characteristics of the NPs (Mayordomo et al., 2025). It is the dynamic interface that determines NP’s biodistribution, cellular absorption, and immune recognition (Panico et al., 2022). However, to create NPs with specific biological effects, further research is needed. (Abaszadeh et al., 2023). In addition, longitudinal studies are also needed to be carried to comprehensive safety and biodegradability of NPs (Kurul et al., 2025). In-depth knowledge regarding non-degradable nanocarriers (such as some inorganic NPs) is very limited, even though nanomaterials like PLGA are thought to be safe (Saker et al., 2024).

Further, intelligent multipurpose NP supported systems may emerge (Kamaraj et al., 2025) in vascular surgeries. These nanoplatforms will combine structural support, targeted drug administration, and sensing into a single cohesive system (Gupta et al., 2025). Smart nano-engineered vascular grafts as conduits are emerging due to the combined efforts of pharmacology, bioelectronics, and materials science. These conduits can assist in real-time monitoring of hemodynamics, local inflammation or thrombosis, and guided drug delivery (Iraola-Picornell et al., 2025). Further, computational design and artificial intelligence (AI) is also being integrated with nanotechnology (Alexandar et al., 2026). AI and machine learning models can predict NPs interactions with biological pathways, and also assist in screening large nanomaterial libraries for targeted biodistribution or drug delivery profiles (Singh et al., 2026).

Smart implants made with NPs can effectively respond to local stimuli (pH, enzymes, mechanical stress) (Zhang et al., 2026). The precision medicine and precise diagnostics of the surgical intervention site can allow nano-supported implants to be customized as per the patient’s physiological status (Mobarak et al., 2023), like smart and antimicrobial activity bearing grafts, implantation in an immunocompromised host.

## 9 Conclusion

In vascular surgery, nanotechnology promises a significant change from passive, traditional instruments to dynamic, intelligent systems that engage with biology at its most basic level. Nanomaterials solve the fundamental flaws in conventional techniques, from giving surgeons real-time molecular vision during procedures to developing implants that actively promote healing and fending off infection. However, it is difficult to get from laboratory discovery to regular clinical application. It necessitates negotiating changing regulatory environments, guaranteeing long-term safety and biocompatibility, and overcoming major obstacles in manufacturing consistency. The future is in creating increasingly intelligent, multipurpose systems that integrate medicine delivery, sensing, and structural support into smooth, patient-specific solutions. Nanotechnology has the potential to improve the accuracy, durability, and success of vascular therapies by further connecting material science and biological knowledge.

## Glossary

AgNP: Silver Nanoparticles
AI: Artificial Intelligence
BVS: Bioresorbable Vascular Scaffolds
CT: Computed Tomography
DEBs: Drug-Eluting Balloons
DES: Drug-Eluting Stents
DNA: Deoxyribose Nucleic Acid
ECM: Extracellular Matrix
EMA: European Medicines Agency
EPCs: Endothelial Progenitor Cells
EPS: Extracellular Polymeric Substance
ePTFE: Expanded Polytetrafluoroethylene
FDA: Food And Drug Administration
GMP: Good Manufacturing Practice
GO: Graphene Oxide
ICAM-1: Intercellular Adhesion Molecule 1
IL-10: Interleukin-10
IL-6: Interleukin-6
LDL: Low-Density Lipoprotein
MRA: Magnetic Resonance Angiography
MRI: Magnetic Resonance Imaging
mRNA: Messenger RNA
NF-kB: Nuclear Factor Kappa B
PLGA: Polylactic-Co-Glycolic Acid
PSGL-1: P-Selectin Glycoprotein Ligand-1
REDV: Arg-Glu-Asp-Val Peptide
RGD: Arginyl glycyl aspartic Acid
RNA: Ribonucleic Acid
ROS: Reactive Oxygen Species
SPIONs: Superparamagnetic Iron Oxide Nanoparticles
TiO2: The Titanium Dioxide
TiO2-NTs: Titanium Dioxide Nanotubes
TNF: Tumor Necrosis Factor
VCAM-1: Vascular Cell Adhesion Molecule 1
VEGF: Vascular Endothelial Growth Factors
VSMCs: Vascular Smooth Muscle Cells

## References

[B1] Abaszadeh F. Ashoub M. H. Khajouie G. Amiri M. (2023). Nanotechnology development in surgical applications: recent trends and developments. Eur. J. Med. Res. 28 (1), 537. 10.1186/s40001-023-01429-4 38001554 PMC10668503

[B2] Abbasi H. Kouchak M. Mirveis Z. Hajipour F. Khodarahmi M. Rahbar N. (2022). What we need to know about liposomes as drug nanocarriers: an updated review. Adv. Pharmaceutical Bulletin 13 (1), 7–23. 10.34172/apb.2023.009 36721822 PMC9871273

[B3] Akhtar S. Babiker F. Al-Kouh A. F Benter I. (2024). The cardiac toxicity of PAMAM dendrimer drug delivery systems can be attenuated with the adjunct use of cardioprotective agents. Biomol. Biomed. 25 (4), 914–924. 10.17305/bb.2024.10735 39319862 PMC11959388

[B4] Alam I. S. Steinberg I. Vermesh O. van den Berg N. S. Rosenthal E. L. van Dam G. M. (2018). Emerging intraoperative imaging modalities to improve surgical precision. Mol. Imaging Biol. 20 (5), 705–715. 10.1007/s11307-018-1227-6 29916118

[B5] Alexandar A. Pandian M. S. Renuka N. Umarani P. (2026). “AI in nanomaterials: enhancing the efficiency of the applications through advanced intelligence,” in Advanced materials for biomedical devices (London: Taylor & Francis Group), 111–120.

[B6] Aliyev A. Israyilova A. Hasanova U. Gakhramanova Z. Ahmadova A. (2025). Nanotechnology in wound healing: a new frontier in regenerative medicine. Micro 5 (4), 60. 10.3390/micro5040060

[B7] Au J. T. Craig G. Longo V. Zanzonico P. Mason M. Fong Y. (2013). Gold nanoparticles provide bright long-lasting vascular contrast for CT imaging. AJR. Am. J. Roentgenol. 200 (6), 1347–1351. 10.2214/AJR.12.8933 23701074

[B8] Bai H. Wu H. Zhang L. Sun P. Liu Y. Xie B. (2022). Adventitial injection of HA/SA hydrogel loaded with PLGA rapamycin nanoparticle inhibits neointimal hyperplasia in a rat aortic wire injury model. Drug Deliv. Transl. Res. 12 (12), 2950–2959. 10.1007/s13346-022-01158-x 35378720

[B9] Barcena A. J. R. Perez J. V. D. Bernardino M. R. San Valentin E. M. D. Damasco J. A. Klusman C. (2024). Controlled delivery of rosuvastatin or rapamycin through electrospun bismuth nanoparticle-infused perivascular wraps promotes arteriovenous fistula maturation. ACS Appl. Mater. & Interfaces 16 (26), 33159–33168. 10.1021/acsami.4c06042 38912610 PMC11725229

[B10] Bashiri G. Padilla M. S. Swingle K. L. Shepherd S. J. Mitchell M. J. Wang K. (2023). Nanoparticle protein corona: from structure and function to therapeutic targeting. Lab a Chip 23 (6), 1432–1466. 10.1039/d2lc00799a 36655824 PMC10013352

[B11] Bi Y. Xie S. Li Z. Dong S. Teng L. (2025). Precise nanoscale fabrication technologies, the “last mile” of medicinal development. Acta Pharm. Sin. B 15 (5), 2372–2401. 10.1016/j.apsb.2025.03.040 40487646 PMC12145073

[B12] Calligaro K. D. Toursarkissian B. Clagett G. P. Towne J. Hodgson K. Moneta G. (2008). Guidelines for hospital privileges in vascular and endovascular surgery: recommendations of the society for vascular surgery. J. Vasc. Surg. 47 (1), 1–5. 10.1016/j.jvs.2007.10.003 18060729

[B13] Caputo F. Favre G. Borchard G. Calzolai L. Fisicaro P. Frejafon E. (2024). Toward an international standardisation roadmap for nanomedicine. Drug Deliv. Transl. Res. 14 (9), 2578–2588. 10.1007/s13346-024-01646-2 38865038 PMC11291566

[B14] Chen F. Cai W. (2014). Tumor vasculature targeting: a generally applicable approach for functionalized nanomaterials. Small 10 (10), 1887–1893. 10.1002/smll.201303627 24591109 PMC4126500

[B15] Chnari E. Nikitczuk J. S. Uhrich K. E. Moghe P. V. (2006). Nanoscale anionic macromolecules can inhibit cellular uptake of differentially oxidized LDL. Biomacromolecules 7 (2), 597–603. 10.1021/bm0506905 16471936

[B16] Cho S. Rasoulianboroujeni M. Kang R. H. Kwon G. S. (2025). From conventional to next-generation strategies: recent advances in polymeric micelle preparation for drug delivery. Pharmaceutics 17 (10), 1360. 10.3390/pharmaceutics17101360 41155995 PMC12566873

[B17] Choi K.-A. Kim J. H. Ryu K. Kaushik N. (2022). Current nanomedicine for targeted vascular disease treatment: trends and perspectives. Int. J. Mol. Sci. 23 (20), 12397. 10.3390/ijms232012397 36293254 PMC9604340

[B18] Chunta S. Khongwichit S. Swangphon P. Srisuk M. Lieberzeit P. A. Amatatongchai M. (2025). Hybrid aptamer molecularly imprinted polymer nanoparticles for reducing oxidized low-density lipoprotein internalization by macrophages. ACS Appl. Mater. & Interfaces. 17, 40101–40115. 10.1021/acsami.5c07018 40591889 PMC12278217

[B19] Costa D. Andreucci M. Ielapi N. Serraino G. F. Mastroroberto P. Bracale U. M. (2023). Infection of vascular prostheses: a comprehensive review. Prosthesis 5 (1), 148–166. 10.3390/prosthesis5010012

[B20] Dagdag O. Haldhar R. Quadri T. W. Daoudi W. Berdimurodov E. Kim H. (2024). Nanomaterials and their properties. Am. Chem. Soc. 1469, 2–17. 10.1021/bk-2024-1469.ch002

[B21] Danker W. I. DeAnglis A. Ferko N. Garcia D. Hogan A. (2021). Comparison of fibrin sealants in peripheral vascular surgery: a systematic review and network meta-analysis. Ann. Med. Surg. 61, 161–168. 10.1016/j.amsu.2020.12.003 33425351 PMC7782199

[B22] Das R. Ambardekar V. Bandyopadhyay P. P. (2021). Titanium dioxide and its applications in mechanical, electrical, optical, and biomedical fields. Titanium Dioxide-Advances Appl. 10.5772/intechopen.98805

[B23] Das K. K. Tiwari R. M. Shankar O. Maiti P. Dubey A. K. (2024). Tissue-engineered vascular grafts for cardiovascular disease management: current strategies, challenges, and future perspectives. Biomaterials Appl. 3 (3), e88. 10.1002/mba2.88

[B24] Davronova G. Siradjitdinova N. Munira K. Khamraev O. Umarova F. Askarov I. (2025). Optical nanobiosensing of cardiovascular disease. Clin. Chim. Acta 582, 120800. 10.1016/j.cca.2025.120800 41422853

[B25] de la Harpe K. M. Kondiah P. P. D. Choonara Y. E. Marimuthu T. du Toit L. C. Pillay V. (2019). The hemocompatibility of nanoparticles: a review of cell-nanoparticle interactions and hemostasis. Cells 8 (10). 10.3390/cells8101209 31591302 PMC6829615

[B26] Díez-Pascual A. M. (2021). Carbon-based nanomaterials. Int. J. Mol. Sci. 22 (14), 7726. 10.3390/ijms22147726 34299346 PMC8307333

[B27] Ding Y. Fu R. Collins C. P. Yoda S. F. Sun C. Ameer G. A. (2022). 3D‐printed radiopaque bioresorbable stents to improve device visualization. Adv. Healthcare Materials 11 (23), 2201955. 10.1002/adhm.202201955 36168854 PMC9742307

[B28] Du J. Wang J. Xu T. Yao H. Yu L. Huang D. (2023). Hemostasis strategies and recent advances in nanomaterials for hemostasis. Mol. Basel, Switz. 28 (13), 5264. 10.3390/molecules28135264 37446923 PMC10343471

[B29] Dube E. Okuthe G. E. (2025). Silver nanoparticle-based antimicrobial coatings: sustainable strategies for microbial contamination control. Microbiol. Res. 16 (6), 110. 10.3390/microbiolres16060110

[B30] Dvorak H. F. (2015). Tumor stroma, tumor blood vessels, and antiangiogenesis therapy. Cancer J. 21 (4), 237–243. 10.1097/PPO.0000000000000124 26222073

[B31] El-Sayed S. M. Youssef A. M. (2023). Eco-friendly biodegradable nanocomposite materials and their recent use in food packaging applications: a review. Sustain. Food Technol. 1 (2), 215–227. 10.1039/d2fb00021k

[B32] Emonde C. K. Eggers M.-E. Wichmann M. Hurschler C. Ettinger M. Denkena B. (2024). Radiopacity enhancements in polymeric implant biomaterials: a comprehensive literature review. ACS Biomaterials Science & Engineering 10 (3), 1323–1334. 10.1021/acsbiomaterials.3c01667 38330191 PMC10934286

[B33] Estelrich J. Sánchez-Martín M. J. Busquets M. A. (2015). Nanoparticles in magnetic resonance imaging: from simple to dual contrast agents. Int. J. Nanomedicine 10, 1727–1741. 10.2147/IJN.S76501 25834422 PMC4358688

[B34] Evans C. E. Iruela-Arispe M. L. Zhao Y.-Y. (2021). Mechanisms of endothelial regeneration and vascular repair and their application to regenerative medicine. Am. J. Pathology 191 (1), 52–65. 10.1016/j.ajpath.2020.10.001 33069720 PMC7560161

[B35] Falk E. Shah P. K. Fuster V. (1995). Coronary plaque disruption. Circulation 92 (3), 657–671. 10.1161/01.cir.92.3.657 7634481

[B36] Fang S. Ellman D. G. Andersen D. C. (2021). Tissue engineering of small-diameter vascular grafts and their *in vivo* evaluation in large animals and humans. Cells 10 (3), 713. 10.3390/cells10030713 33807009 PMC8005053

[B37] Fernandes C. Jathar M. Sawant B. K. S. Warde T. (2023). “Scale-up of nanoparticle manufacturing process,” in Pharmaceutical process engineering and scale-up principles (Springer), 173–203.

[B38] Fodor M. Fodor L. Bota O. (2021). The role of nanomaterials and nanostructured surfaces for improvement of biomaterial peculiarities in vascular surgery: a review. Part. Sci. Technol. 39 (8), 944–953. 10.1080/02726351.2021.1871692

[B39] Gaudino M. Antoniades C. Benedetto U. Deb S. Di Franco A. Di Giammarco G. (2017). Mechanisms, consequences, and prevention of coronary graft failure. Circulation 136 (18), 1749–1764. 10.1161/CIRCULATIONAHA.117.027597 29084780

[B40] Ghazi R. Ibrahim T. K. Nasir J. A. Gai S. Ali G. Boukhris I. (2025). Iron oxide based magnetic nanoparticles for hyperthermia, MRI and drug delivery applications: a review. RSC Advances 15 (15), 11587–11616. 10.1039/d5ra00728c 40230636 PMC11995399

[B41] Gheorghiță D. Moldovan H. Robu A. Bița A.-I. Grosu E. Antoniac A. (2023). Chitosan-based biomaterials for hemostatic applications: a review of recent advances. Int. J. Mol. Sci. 24 (13), 10540. 10.3390/ijms241310540 37445718 PMC10342007

[B42] Guo Y. Wang M. Liu Q. Liu G. Wang S. Li J. (2023). Recent advances in the medical applications of hemostatic materials. Theranostics 13 (1), 161–196. 10.7150/thno.79639 36593953 PMC9800728

[B43] Gupta S. Urs T. J. Aggarwal N. Sen S. Bondhopadhyay B. (2025). The potential of next-generation multi-functional nanoplatforms for breast cancer. Anti-Cancer Agents Med. Chem. 10.2174/0118715206392103250715115020 40734427

[B44] Hachhach M. Bayou S. El Kasmi A. Saidi M. Z. Akram H. Hanafi M. (2025). Towards sustainable Scaling-Up of nanomaterials fabrication: current situation, challenges, and future perspectives. Eng 6 (7), 149. 10.3390/eng6070149

[B45] Hajipour M. J. Safavi‐Sohi R. Sharifi S. Mahmoud N. Ashkarran A. A. Voke E. (2023). An overview of nanoparticle protein corona literature. Small 19 (36), 2301838. 10.1002/smll.202301838 37119440 PMC10552659

[B46] HalvaeiKhanekahdani P. Wu Y. Ta H. T. (2025). miRNAs in cardiovascular disease and an update on emerging trend in electrochemical biosensors for miRNA detection. Crit. Rev. Biotechnol. 46, 1–28. 10.1080/07388551.2025.2584689 41276298

[B47] Haririan Y. Elahi A. Shadman-Manesh V. Rezaei H. Mohammadi M. Asefnejad A. (2025). Advanced nanostructured biomaterials for accelerated wound healing: insights into biological interactions and therapeutic innovations: a comprehensive review. Mater. & Des. 258, 114698. 10.1016/j.matdes.2025.114698

[B48] Hashemzadeh S. Bina F. Mirkamali Khounsari H. (2024). Nanotechnology in the development of cardiac stents. J. Drug Deliv. Sci. Technol. 95, 105596. 10.1016/j.jddst.2024.105596

[B49] Hellmund K. S. Koksch B. (2019). Self-assembling peptides as extracellular matrix mimics to influence stem cell’s fate. Front. Chem. 7, 172. 10.3389/fchem.2019.00172 31001512 PMC6455064

[B50] Herdiana Y. (2025). Bridging the gap: the role of advanced formulation strategies in the clinical translation of nanoparticle-based drug delivery systems. Int. J. Nanomedicine 20, 13039–13053. 10.2147/IJN.S554821 41185748 PMC12579833

[B51] Hernandez-Sanchez D. Comtois-Bona M. Muñoz M. Ruel M. Suuronen E. J. Alarcon E. I. (2024). Manufacturing and validation of small-diameter vascular grafts: a mini review. IScience 27 (6), 109845. 10.1016/j.isci.2024.109845 38799581 PMC11126982

[B52] Hickman D. A. Pawlowski C. L. Sekhon U. D. S. Marks J. Gupta A. S. (2018). Biomaterials and advanced technologies for hemostatic management of bleeding. Adv. Mater. 30 (4), 1700859. 10.1002/adma.201700859 29164804 PMC5831165

[B53] Holmstedt C. A. Turan T. N. Chimowitz M. I. (2013). Atherosclerotic intracranial arterial stenosis: risk factors, diagnosis, and treatment. Lancet Neurology 12 (11), 1106–1114. 10.1016/S1474-4422(13)70195-9 24135208 PMC4005874

[B54] Iraola-Picornell G. Berastegui E. Castells-Sala C. Martorell J. Bayes-Genis A. Muñoz-Guijosa C. (2025). A narrative review of vascular conduits for coronary artery bypass grafting. Cell Transplant. 34, 9636897251407160. 10.1177/09636897251407160 41437519 PMC12745543

[B55] Islam P. Schaly S. Abosalha A. K. Boyajian J. Thareja R. Ahmad W. (2024). Nanotechnology in development of next generation of stent and related medical devices: current and future aspects. WIREs Nanomedicine Nanobiotechnology 16 (2), e1941. 10.1002/wnan.1941 38528392

[B56] Iyer R. Kuriakose A. E. Yaman S. Su L.-C. Shan D. Yang J. (2019). Nanoparticle eluting-angioplasty balloons to treat cardiovascular diseases. Int. J. Pharm. 554, 212–223. 10.1016/j.ijpharm.2018.11.011 30408532 PMC6489505

[B57] Jacob S. Varkey N. R. Boddu S. H. S. Gorain B. Rao R. Nair A. B. (2025). Advances in lipid-polymer hybrid nanoparticles: design strategies, functionalization, oncological and non-oncological clinical prospects. Pharmaceuticals 18 (12), 1772. 10.3390/ph18121772 41471261 PMC12736075

[B58] Jaffer F. A. Libby P. Weissleder R. (2007). Molecular imaging of cardiovascular disease. Circulation 116 (9), 1052–1061. 10.1161/CIRCULATIONAHA.106.647164 17724271

[B59] Jayagopal A. Russ P. K. Haselton F. R. (2007). Surface engineering of quantum dots for *in vivo* vascular imaging. Bioconjugate Chem. 18 (5), 1424–1433. 10.1021/bc070020r 17760416 PMC2853010

[B60] Jeong Y.-J. Jeong S. Kim S. Kim H. J. Jo J. Shanmugasundaram A. (2023). 3D-printed cardiovascular polymer scaffold reinforced by functional nanofiber additives for tunable mechanical strength and controlled drug release. Chem. Eng. J. 454, 140118. 10.1016/j.cej.2022.140118

[B61] Jiang W. Rutherford D. Vuong T. Liu H. (2017). Nanomaterials for treating cardiovascular diseases: a review. Bioact. Mater. 2 (4), 185–198. 10.1016/j.bioactmat.2017.11.002 29744429 PMC5935516

[B62] Jiao S. Zhang X. Cai H. Wu S. Ou X. Han G. (2023). Recent advances in biomimetic hemostatic materials. Mater. Today Bio 19, 100592. 10.1016/j.mtbio.2023.100592 36936399 PMC10020683

[B63] Junkar I. Kulkarni M. Drašler B. Rugelj N. Mazare A. Flašker A. (2016). Influence of various sterilization procedures on TiO2 nanotubes used for biomedical devices. Bioelectrochemistry 109, 79–86. 10.1016/j.bioelechem.2016.02.001 26900885

[B64] Kamaraj I. Kamaraj S. Shanmugam G. (2025). Emerging strategies and multifunctional applications of nanomaterials in modern nanomedicine. Med. Nov. Technol. Devices 28, 100400. 10.1016/j.medntd.2025.100400

[B65] Khan S. Hossain M. K. (2022). “2 - classification and properties of nanoparticles,” in Woodhead publishing series in composites science and engineering. Editors Mavinkere Rangappa S. Parameswaranpillai J. Yashas Gowda T. G. Siengchin S. (Amsterdam, Netherlands: Elsevier), 15–54.

[B66] Kilbride B. F. Narsinh K. H. Jordan C. D. Mueller K. Moore T. Martin A. J. (2022). MRI-guided endovascular intervention: current methods and future potential. Expert Review Medical Devices 19 (10), 763–778. 10.1080/17434440.2022.2141110 36373162 PMC9869980

[B67] Klusman C. Martin B. Perez J. V. D. Barcena A. R. Bernardino M. R. Valentin E. M. D. S. (2023). Rosuvastatin-eluting gold-nanoparticle-loaded perivascular wrap for enhanced arteriovenous fistula maturation in a murine model. Adv. Fiber Mater. 5 (6), 1986–2001. 10.1007/s42765-023-00315-2

[B68] Kosuge H. Nakamura M. Oyane A. Tajiri K. Murakoshi N. Sakai S. (2022). Potential of gold nanoparticles for noninvasive imaging and therapy for vascular inflammation. Mol. Imaging Biol. 24 (5), 692–699. 10.1007/s11307-021-01654-5 34580810 PMC9581827

[B69] Kumar D. Amika Kumar D. Singh P. Chauhan A. S. Kapoor S. (2025). Polymer/carbon nanotube composites: a comprehensive review on fabrication techniques and their consequences. AIMS Mater. Sci. 12 (4), 813–844. 10.3934/matersci.2025035

[B70] Kurul F. Turkmen H. Cetin A. E. Topkaya S. N. (2025). Nanomedicine: how nanomaterials are transforming drug delivery, bio-imaging, and diagnosis. Next Nanotechnol. 7, 100129. 10.1016/j.nxnano.2024.100129

[B71] Kuznetsov K. A. Murashov I. S. Chernonosova V. S. Chelobanov B. P. Stepanova A. O. Sergeevichev D. S. (2020). Vascular stents coated with electrospun drug-eluting material: functioning in rabbit iliac artery. Polymers 12 (8). 10.3390/polym12081741 32759856 PMC7465440

[B72] Larguinho M. Figueiredo S. Cordeiro A. Carlos F. F. Cordeiro M. Pedrosa P. (2015). “Nanoparticles for diagnostics and imaging,” in Frontiers in nanomedicine (Sharjah, United Arab Emirates (UAE): Bentham Science Publishers), 3–46.

[B73] Li X. Wu M. Li J. Guo Q. Zhao Y. Zhang X. (2022). Advanced targeted nanomedicines for vulnerable atherosclerosis plaque imaging and their potential clinical implications. Front. Pharmacol. 13, 906512. 10.3389/fphar.2022.906512 36313319 PMC9606597

[B74] Li X. Yue R. Guan G. Zhang C. Zhou Y. Song G. (2023). Recent development of pH-responsive theranostic nanoplatforms for magnetic resonance imaging-guided cancer therapy. Explor. (Beijing, China) 3 (3), 20220002. 10.1002/EXP.20220002 37933379 PMC10624388

[B75] Li Q. Ding X. Chen C. Zhang K. Dong R. (2025). An overview of small diameter vascular grafts: from materials to fabrication. Mater. Adv. 6, 6221–6242. 10.1039/d5ma00663e

[B76] Li S. Wang J. Zhang H. Zhang X. (2025). Advances in graphene oxide-based polymeric wound dressings for wound healing. Front. Mater. 12, 1635502. 10.3389/fmats.2025.1635502

[B77] Liu X. Wang N. Liu X. Deng R. Kang R. Xie L. (2022). Vascular repair by grafting based on magnetic nanoparticles. Pharmaceutics 14 (7), 1433. 10.3390/pharmaceutics14071433 35890328 PMC9320478

[B78] Liu B. Wang Y. Gong W. Han S. Lv Z. Zhang Z. (2025). Natural, engineered, and hybrid platelet membrane–based nanotherapeutics for inflammatory diseases. Int. J. Nanomedicine 20, 14149–14184. 10.2147/IJN.S558928 41332500 PMC12666424

[B79] Liu Y. Li Y. Chen J. Xie P. Yin Z. (2025). Construction of fibrin-targeted nanoparticles for imaging diagnosis and treatment of arterial thrombosis. Nanoscale 17 (12), 7351–7366. 10.1039/d4nr05377j 39992663

[B80] Luo X. Pang Z. Li J. Anh M. Kim B. S. Gao G. (2024). Bioengineered human arterial equivalent and its applications from vascular graft to *in vitro* disease modeling. IScience 27 (11), 111215. 10.1016/j.isci.2024.111215 39555400 PMC11565542

[B81] Ma X. Tian Y. Yang R. Wang H. Allahou L. W. Chang J. (2024). Nanotechnology in healthcare, and its safety and environmental risks. J. Nanobiotechnology 22 (1), 715. 10.1186/s12951-024-02901-x 39548502 PMC11566612

[B82] MacRitchie N. Di Francesco V. (2021). “Nanoparticle theranostics in cardiovascular inflammation,” in Seminars in immunology (Elsevier). 10.1016/j.smim.2021.101536 PMC8811479 34862118

[B83] Manisekaran R. Chettiar A. R. Marasamy L. Arthikala M. Kandasamy G. Ibarra V. C. (2025). Copper, zinc, and titanium‐based semiconductor nanomaterials for antimicrobial coatings and their mechanisms. Nano Sel. 6 (5), e202400155. 10.1002/nano.202400155

[B84] Mariappan N. (2019). Recent trends in nanotechnology applications in surgical specialties and orthopedic surgery. Biomed. Pharmacol. J. 12 (3), 1095–1127. 10.13005/bpj/1739

[B85] Materón E. M. Miyazaki C. M. Carr O. Joshi N. Picciani P. H. S. Dalmaschio C. J. (2021). Magnetic nanoparticles in biomedical applications: a review. Appl. Surf. Sci. Adv. 6, 100163. 10.1016/j.apsadv.2021.100163

[B86] Matoba T. Egashira K. (2014). Nanoparticle-mediated drug delivery system for cardiovascular disease. Int. Heart J. 55 (4), 281–286. 10.1536/ihj.14-150 24942639

[B87] Matsui A. Lee B. T. Winer J. H. Vooght C. S. Laurence R. G. Frangioni J. V. (2009). Real-time intraoperative near-infrared fluorescence angiography for perforator identification and flap design. Plastic Reconstr. Surg. 123 (3), 125e–127e. 10.1097/PRS.0b013e31819a3617 19319038

[B88] Mayordomo N. M. Zatarain-Beraza A. Valerio F. Álvarez-Méndez V. Turegano P. Herranz-García L. (2025). The protein Corona paradox: challenges in achieving true biomimetics in nanomedicines. Biomimetics 10 (5), 276. 10.3390/biomimetics10050276 40422106 PMC12108862

[B89] Melchiorri A. J. Hibino N. Yi T. Lee Y. U. Sugiura T. Tara S. (2015). Contrasting biofunctionalization strategies for the enhanced endothelialization of biodegradable vascular grafts. Biomacromolecules 16 (2), 437–446. 10.1021/bm501853s 25545620 PMC4325601

[B90] Meng X. Liu A. Wu C. Han X. Yang Q. Qiu H. (2025). Research progress of magnesium alloys and its alloys in medical applications. Int. J. General Med. 18, 7101–7126. 10.2147/IJGM.S565096 41323101 PMC12664577

[B91] Mishra A. Aggarwal A. Khan F. (2024). Medical device-associated infections caused by biofilm-forming microbial pathogens and controlling strategies. Antibiotics 13 (7), 623. 10.3390/antibiotics13070623 39061305 PMC11274200

[B92] Miyasato D. L. Mohamed A. W. Zavaleta C. (2021). A path toward the clinical translation of nano-based imaging contrast agents. WIREs Nanomedicine Nanobiotechnology 13 (6), e1721. 10.1002/wnan.1721 33938151

[B93] Mobarak M. H. Islam M. A. Hossain N. Al Mahmud M. Z. Rayhan M. T. Nishi N. J. (2023). Recent advances of additive manufacturing in implant fabrication – a review. Appl. Surf. Sci. Adv. 18, 100462. 10.1016/j.apsadv.2023.100462

[B94] Mozaffari H. Javadi P. Faridi-Majidi R. Derakhshan M. A. (2025). Gold nanoparticle-incorporated polyurethane/collagen nanofibrous scaffold as a potential cardiac patch. J. Bionic Eng. 22, 1–16. 10.1007/s42235-025-00705-9

[B95] Muñoz J. M. (2018). Chemical design and validation of Ca2+-releasing platforms to promote vascularization in tissue regeneration. Barcelona, Spain: Universitat Politècnica de Catalunya (UPC) – Servei de Biblioteques, Publicacions i Arxius.

[B96] Murphy D. Aghayev A. Steigner M. L. (2018). Vascular CT and MRI: a practical guide to imaging protocols. Insights Into Imaging 9 (2), 215–236. 10.1007/s13244-018-0597-2 29541955 PMC5893493

[B97] Naeim H. Mahdavian F. Rodrigue D. (2025). Review on electrospinning of recycled polymer-derived fibers: a road towards sustainability, production and applications. J. Environ. Chem. Eng. 13 (6), 119283. 10.1016/j.jece.2025.119283

[B98] Nahrendorf M. Sosnovik D. E. French B. A. Swirski F. K. Bengel F. Sadeghi M. M. (2009). Multimodality cardiovascular molecular imaging, part II. Circ. Cardiovasc. Imaging 2 (1), 56–70. 10.1161/CIRCIMAGING.108.839092 19808565 PMC2760054

[B99] Naskar A. Kilari S. Baranwal G. Kane J. Misra S. (2024). Nanoparticle-based drug delivery for vascular applications. Bioengineering 11 (12), 1222. 10.3390/bioengineering11121222 39768040 PMC11673055

[B100] Nott D. M. (2022). 27 - vascular surgery in the austere environment. 332–351. Elsevier.

[B101] Palmerston Mendes L. Pan J. Torchilin V. P. (2017). Dendrimers as nanocarriers for nucleic acid and drug delivery in cancer therapy. Molecules 22 (9), 1401. 10.3390/molecules22091401 28832535 PMC5600151

[B102] Panico S. Capolla S. Bozzer S. Toffoli G. Dal Bo M. Macor P. (2022). Biological features of nanoparticles: protein Corona formation and interaction with the immune system. Pharmaceutics 14 (12), 2605. 10.3390/pharmaceutics14122605 36559099 PMC9781747

[B103] Pawelec K. M. Hix J. M. Troia A. MacRenaris K. W. Kiupel M. Shapiro E. M. (2024). *In vivo* micro-computed tomography evaluation of radiopaque, polymeric device degradation in normal and inflammatory environments. Acta Biomater. 181, 222–234. 10.1016/j.actbio.2024.04.031 38648912 PMC11144086

[B104] Rani A. Marsche G. (2023). A current update on the role of HDL-based nanomedicine in targeting macrophages in cardiovascular disease. Pharmaceutics 15 (5), 1504. 10.3390/pharmaceutics15051504 37242746 PMC10221824

[B105] Ricco J.-B. Assadian O. (2011). Antimicrobial silver grafts for prevention and treatment of vascular graft infection. Seminars Vasc. Surg. 24 (4), 234–241. 10.1053/j.semvascsurg.2011.10.006 22230679

[B106] Rickel A. P. Deng X. Engebretson D. Hong Z. (2021). Electrospun nanofiber scaffold for vascular tissue engineering. Mater. Sci. & Eng. 129, 112373. 10.1016/j.msec.2021.112373 34579892 PMC8486306

[B107] Rogers T. Campbell-Washburn A. E. Ramasawmy R. Yildirim D. K. Bruce C. G. Grant L. P. (2023). Interventional cardiovascular magnetic resonance: state-of-the-art. J. Cardiovasc. Magnetic Reson. 25 (1), 48. 10.1186/s12968-023-00956-7 37574552 PMC10424337

[B108] Roguska A. Belcarz A. Piersiak T. Pisarek M. Ginalska G. Lewandowska M. (2012). Evaluation of the antibacterial activity of Ag-Loaded TiO2 nanotubes. Eur. J. Inorg. Chem. 2012, 5199–5206. 10.1002/ejic.201200508

[B109] Roy S. Das A. Chakraborty T. Bhattacharya B. (2023). “Chapter 5 - nanotechnology-based regenerative approaches,” in Nanotechnology in biomedicine. Editors Mondal A. Nayak A. K. Chakraborty P. B. (Elsevier), 181–280.

[B110] Sahoo J. Sarkhel S. Mukherjee N. Jaiswal A. (2022). Nanomaterial-based antimicrobial coating for biomedical implants: new age solution for biofilm-associated infections. ACS Omega 7 (50), 45962–45980. 10.1021/acsomega.2c06211 36570317 PMC9773971

[B111] Sakamoto A. Jinnouchi H. Torii S. Virmani R. Finn A. V. (2018). Understanding the impact of stent and scaffold material and strut design on coronary artery thrombosis from the basic and clinical points of view. Bioengineering 5 (3), 71. 10.3390/bioengineering5030071 30181463 PMC6164756

[B112] Saker R. Regdon G. Sovány T. (2024). Pharmacokinetics and toxicity of inorganic nanoparticles and the physicochemical properties/factors affecting them. J. Drug Deliv. Sci. Technol. 99, 105979. 10.1016/j.jddst.2024.105979

[B113] Scimeca J. Verron E. (2022). Nano-engineered biomaterials: safety matters and toxicity evaluation. Mater. Today Adv. 15, 100260. 10.1016/j.mtadv.2022.100260

[B114] Sharifi S. Mahmoud N. N. Voke E. Landry M. P. Mahmoudi M. (2022). Importance of standardizing analytical characterization methodology for improved reliability of the nanomedicine literature. Nano-micro Letters 14 (1), 172. 10.1007/s40820-022-00922-5 35987931 PMC9392440

[B115] Sharma N. Chauhan V. Singh S. Kumar P. Verma S. Gupta S. (2025). A spotlight on PLGA-based nanoparticles: pioneering a new era in the therapeutics of cardiovascular disorders. Curr. Pharm. Des. 31 (41), 3267–3284. 10.2174/0113816128361869250409171305 40325566

[B116] Shaw S. Y. (2009). Molecular imaging in cardiovascular disease: targets and opportunities. Nat. Rev. Cardiol. 6 (9), 569–579. 10.1038/nrcardio.2009.119 19621013

[B117] Shen S. Koonjoo N. Boele T. Lu J. Waddington D. E. J. Zhang M. (2024). Enhancing organ and vascular contrast in preclinical ultra-low field MRI using superparamagnetic iron oxide nanoparticles. Commun. Biol. 7 (1), 1197. 10.1038/s42003-024-06884-1 39342051 PMC11438998

[B118] Singh R. Srinivas S. P. Kumawat M. Daima H. K. (2024). Ligand-based surface engineering of nanomaterials: trends, challenges, and biomedical perspectives. OpenNano 15, 100194. 10.1016/j.onano.2023.100194

[B119] Singh A. K. Sharma R. Kujur V. S. Poddar M. Kumar A. Kumar S. (2025). Integration of nanotechnology and nanomaterials in biomaterials research. Biomater. Connect. 2 (1), 1–10. 10.69709/biomatc.2025.188313

[B120] Singh V. Sinha S. Verma J. (2026). Bioinformatics models in drug delivery: predicting biomaterial-biological interactions for targeted therapies. Next Nanotechnol. 9, 100335. 10.1016/j.nxnano.2025.100335

[B121] Snyder B. (2015). New nanoparticle enhances success rate of coronary artery bypass grafts. Available online at: https://news.vumc.org/June 18, 2015/new-nanoparticle- enhances-success-rate-of-coronary-artery-bypass-grafts/.

[B122] Su Q. Xue Y. Wang C. Zhou Q. Zhao Y. Su J. (2025). Strategies and applications of antibacterial surface-modified biomaterials. Bioact. Mater. 53, 114–140. 10.1016/j.bioactmat.2025.07.009 40688018 PMC12274879

[B123] Sun B. Slomberg D. L. Chudasama S. L. Lu Y. Schoenfisch M. H. (2012). Nitric oxide-releasing dendrimers as antibacterial agents. Biomacromolecules 13 (10), 3343–3354. 10.1021/bm301109c 23013537 PMC3482834

[B124] Sun M. Miyazawa K. Pendekanti T. Razmi A. Firlar E. Yang S. (2020). Combination targeting of ‘platelets+ fibrin’enhances clot anchorage efficiency of nanoparticles for vascular drug delivery. Nanoscale 12 (41), 21255–21270. 10.1039/d0nr03633a 33063812 PMC8112300

[B125] Tange F. P. van den Hoven P. van Schaik J. Schepers A. van der Bogt K. E. A. van Rijswijk C. S. P. (2024). Near-infrared fluorescence imaging with indocyanine green to predict clinical outcome after revascularization in lower extremity arterial disease. Angiology 75 (9), 884–892. 10.1177/00033197231186096 37358400 PMC11375904

[B126] Tian L. Lee P. Singhana B. Chen A. Qiao Y. Lu L. (2017). Radiopaque resorbable inferior vena cava filter infused with gold nanoparticles. Sci. Rep. 7 (1), 2147. 10.1038/s41598-017-02508-3 28526874 PMC5438341

[B127] Toong D. W. Y. Toh H. W. Ng J. C. K. Wong P. E. H. Leo H. L. Venkatraman S. (2020). Bioresorbable polymeric scaffold in cardiovascular applications. Int. J. Mol. Sci. 21 (10), 3444. 10.3390/ijms21103444 32414114 PMC7279389

[B128] Toong D. W. Y. Ng J. C. K. Cui F. Leo H. L. Zhong L. Lian S. S. (2022). Nanoparticles-reinforced poly-l-lactic acid composite materials as bioresorbable scaffold candidates for coronary stents: insights from mechanical and finite element analysis. J. Mech. Behav. Biomed. Mater. 125, 104977. 10.1016/j.jmbbm.2021.104977 34814078

[B129] Tsai C.-C. Chang Y. Sung H.-W. Hsu J.-C. Chen C.-N. (2001). Effects of heparin immobilization on the surface characteristics of a biological tissue fixed with a naturally occurring crosslinking agent (genipin): an *in vitro* study. Biomaterials 22 (6), 523–533. 10.1016/s0142-9612(00)00206-4 11219715

[B130] Vairaperumal T. Huang C.-C. Liu P. Y. (2023). Optical nanobiosensor-based point-of-care testing for cardiovascular disease biomarkers. ACS Appl. Bio Mater. 6 (7), 2591–2613. 10.1021/acsabm.3c00223 37317822

[B131] Wang S. He H. Mao Y. Zhang Y. Gu N. (2024). Advances in atherosclerosis theranostics harnessing iron oxide‐based nanoparticles. Adv. Sci. 11 (17), 2308298. 10.1002/advs.202308298 38368274 PMC11077671

[B132] Wang Y. Xu Y. Song J. Liu X. Liu S. Yang N. (2024). Tumor cell-targeting and tumor microenvironment–responsive nanoplatforms for the multimodal imaging-guided photodynamic/photothermal/chemodynamic treatment of cervical cancer. Int. J. Nanomedicine 19, 5837–5858. 10.2147/IJN.S466042 38887692 PMC11182360

[B133] Wang L. Periyasami G. Aldalbahi A. Fogliano V. (2021). The antimicrobial activity of silver nanoparticles biocomposite films depends on the silver ions release behaviour. Food Chem. 359, 129859. 10.1016/j.foodchem.2021.129859 33957323

[B134] Wang Z. Wang X. Wan J. Xu F. Zhao N. Chen M. (2021). Optical imaging in the second near infrared window for vascular bioimaging. Small 17 (43), 2103780. 10.1002/smll.202103780 34643028

[B135] Wu B. Du F. A W. Li G. Wang X. (2022). Graphene-based hemostatic sponge. Chin. Chem. Lett. 33 (2), 703–713. 10.1016/j.cclet.2021.06.029

[B136] Wu Y. Tran H. D. N. Sajin D. Moonshi S. S. Fithri N. A. Kurniawan N. (2025). Vascular-protective effects of cerium oxide nanoparticles complexed with iron oxide and methotrexate: reversing atherosclerosis in a murine model. ACS Nano Med. 1, 250–267. 10.1021/acsnanomed.5c00036

[B137] Xie G. Lin S. Wu F. Liu J. (2023). Nanomaterial-based ophthalmic drug delivery. Adv. Drug Deliv. Rev. 200, 115004. 10.1016/j.addr.2023.115004 37433372

[B138] Xu H. Li S. Liu Y. S. (2022). Nanoparticles in the diagnosis and treatment of vascular aging and related diseases. Signal Transduction Targeted Therapy 7 (1), 231. 10.1038/s41392-022-01082-z 35817770 PMC9272665

[B139] Xu B. Li S. Shi R. Liu H. (2023). Multifunctional mesoporous silica nanoparticles for biomedical applications. Signal Transduction Targeted Therapy 8 (1), 435. 10.1038/s41392-023-01654-7 37996406 PMC10667354

[B140] Yadav H. Malviya R. Kaushik N. (2024). Chitosan in biomedicine: a comprehensive review of recent developments. Carbohydr. Polym. Technol. Appl. 8, 100551. 10.1016/j.carpta.2024.100551

[B141] Yang F. Xue J. Wang G. Diao Q. (2022). Nanoparticle-based drug delivery systems for the treatment of cardiovascular diseases. Front. Pharmacol. 13, 999404. 10.3389/fphar.2022.999404 36172197 PMC9512262

[B142] Yang J. Zeng H. Luo Y. Chen Y. Wang M. Wu C. (2024). Recent applications of PLGA in drug delivery systems. Polymers 16 (18), 2606. 10.3390/polym16182606 39339068 PMC11435547

[B143] Yao Y. Pohan G. Cutiongco M. F. A. Jeong Y. Kunihiro J. Zaw A. M. (2023). *In vivo* evaluation of compliance mismatch on intimal hyperplasia formation in small diameter vascular grafts. Biomaterials Sci. 11 (9), 3297–3307. 10.1039/d3bm00167a 36943136 PMC10160004

[B144] Zabaglo M. Leslie S. W. Sharman T. (2024). “Postoperative wound infections,” in StatPearls (Treasure Island, FL: StatPearls Publishing). 32809368

[B145] Zeng J. Zhang Y. (2025). Biomimetic ginsenoside Rb1 and probucol Co-Assembled nanoparticles for targeted atherosclerosis therapy via inhibition of oxidative stress, inflammation, and lipid deposition. ACS Nano. 19, 22968–22987. 10.1021/acsnano.5c02492 40534137

[B146] Zeng L. Wei Y. Qiu Y. Bi R. Peng H. Hu B. (2025). Nanomaterial-based anti-angiogenic gene therapy for retinal neovascular diseases: mechanistic insights and preclinical advances. Int. J. Nanomedicine 20, 11361–11388. 10.2147/IJN.S521960 40989889 PMC12452977

[B147] Zhang P. Li Y. Li X. Wang Y. Lin H. Zhang N. (2024). Shedding light on vascular imaging: the revolutionary role of nanotechnology. J. Nanobiotechnology 22 (1), 757. 10.1186/s12951-024-03042-x 39695727 PMC11657597

[B148] Zhang N. Zhu Y. Li F. Wang Y. Fu Z. Jia M. (2025). Research progress on nanomaterial-empowered electrochemical biosensors for the detection of cardiac troponin I. RSC Advances 15 (32), 26473–26489. 10.1039/d5ra04555j 40703066 PMC12285579

[B149] Zhang P. Nie Y. Wang X. Zhang X. Liu L. (2026). Next-generation smart ophthalmic biomaterials: from passive response to active interaction and closed-loop control. Bioact. Mater. 56, 522–558. 10.1016/j.bioactmat.2025.10.037 41234295 PMC12607147

[B150] Zhao A. Sun J. Liu Y. (2023). Understanding bacterial biofilms: from definition to treatment strategies. Front. Cell. Infect. Microbiol. 13, 1137947. 10.3389/fcimb.2023.1137947 37091673 PMC10117668

[B151] Zhao X. Chen W. Wu J. Shen Y. Xu B. Chen Z. (2025). Application of biomimetic cell membrane-coated nanocarriers in cardiovascular diseases. Int. J. Nanomedicine 20, 8249–8289. 10.2147/IJN.S531558 40589723 PMC12208305

[B152] Zhen J. Li X. Yu H. Du B. (2024). High-density lipoprotein mimetic nano-therapeutics targeting monocytes and macrophages for improved cardiovascular care: a comprehensive review. J. Nanobiotechnology 22 (1), 263. 10.1186/s12951-024-02529-x 38760755 PMC11100215

[B153] Zheng C. Liu J. Bai Q. Quan Y. Li Z. Chen W. (2022). Preparation and hemostatic mechanism of bioactive glass-based membrane-like structure camouflage composite particles. Mater. & Des. 223, 111116. 10.1016/j.matdes.2022.111116

[B154] Zhou Z. Tian R. Wang Z. Yang Z. Liu Y. Liu G. (2017). Artificial local magnetic field inhomogeneity enhances T2 relaxivity. Nat. Commun. 8 (1), 15468. 10.1038/ncomms15468 28516947 PMC5454366

[B155] Zizhou R. Wang X. Houshyar S. (2022). Review of polymeric biomimetic small-diameter vascular grafts to tackle intimal hyperplasia. ACS Omega 7 (26), 22125–22148. 10.1021/acsomega.2c01740 35811906 PMC9260943

[B156] Zulkifli M. Z. Nordin D. Shaari N. Kamarudin S. K. (2023). Overview of electrospinning for tissue engineering applications. Polymers 15 (11), 2418. 10.3390/polym15112418 37299217 PMC10255387

