# Supplementary figures and images for: Advancements in nanomaterials for the treatment and management of vascular surgeries: from drug delivery to biomedical implants
Source: Front Bioeng Biotechnol. 2026 Mar 24;14:1788897. doi: 10.3389/fbioe.2026.1788897 (PMC13055620; doi:10.3389/fbioe.2026.1788897)

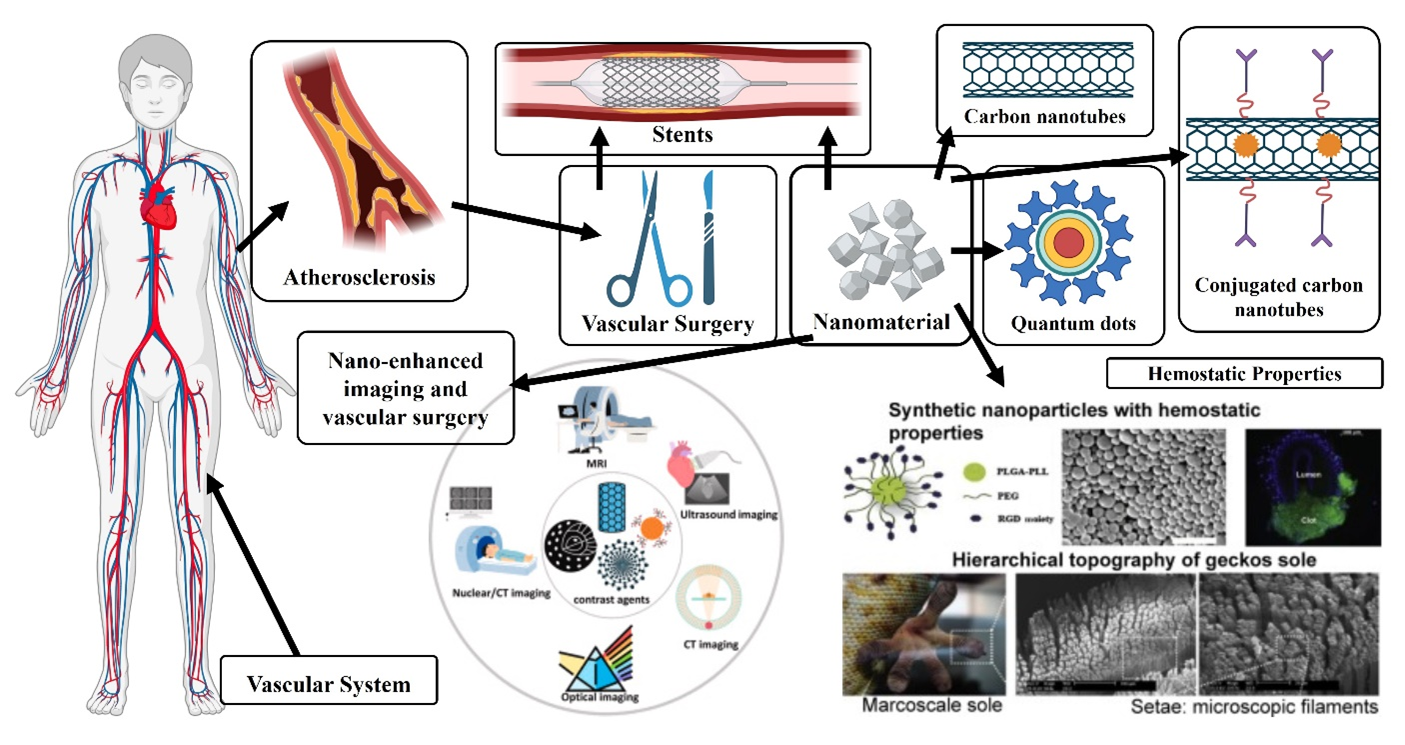

Supplement: Supplementary file 1 [file Image1.tif]
